# The chiropractic profession: a scoping review of utilization rates, reasons for seeking care, patient profiles, and care provided

**DOI:** 10.1186/s12998-017-0165-8

**Published:** 2017-11-22

**Authors:** Peter J. H. Beliveau, Jessica J. Wong, Deborah A. Sutton, Nir Ben Simon, André E. Bussières, Silvano A. Mior, Simon D. French

**Affiliations:** 1 Department of Public Health Sciences, Queen’s University, Kingston, Canada; 2 UOIT-CMCC Centre for the Study of Disability Prevention and Rehabilitation, University of Ontario Institute of Technology (UOIT) and Canadian Memorial Chiropractic College (CMCC), Toronto, Canada; 3 Department of Research, Canadian Memorial Chiropractic College, 6100 Leslie St, Toronto, ON M2H 3J1 Canada; 4 School of Physical and Occupational Therapy, McGill University, Montréal, Canada; 5 Département chiropratique, Université du Québec à Trois-Rivières, Trois-Rivières, Canada; 6 Centre de recherche interdisciplinaire en réadaptation (CRIR), Montréal, Canada; 7 School of Rehabilitation Therapy, Queen’s University, Kingston, Canada; 8 Department of Chiropractic, Macquarie University, Sydney, Australia

**Keywords:** Chiropractic, utilization, patient demographics, assessment, treatment, statistics, scoping review

## Abstract

**Background:**

Previous research has investigated utilization rates, who sees chiropractors, for what reasons, and the type of care that chiropractors provide. However, these studies have not been comprehensively synthesized. We aimed to give a global overview by summarizing the current literature on the utilization of chiropractic services, reasons for seeking care, patient profiles, and assessment and treatment provided.

**Methods:**

Systematic searches were conducted in MEDLINE, CINAHL, and Index to Chiropractic Literature using keywords and subject headings (MeSH or ChiroSH terms) from database inception to January 2016. Eligible studies: 1) were published in English or French; 2) were case series, descriptive, cross-sectional, or cohort studies; 3) described patients receiving chiropractic services; and 4) reported on the following theme(s): utilization rates of chiropractic services; reasons for attending chiropractic care; profiles of chiropractic patients; or, types of chiropractic services provided. Paired reviewers independently screened all citations and data were extracted from eligible studies. We provided descriptive numerical analysis, e.g. identifying the median rate and interquartile range (e.g., chiropractic utilization rate) stratified by study population or condition.

**Results:**

The literature search retrieved 14,149 articles; 328 studies (reported in 337 articles) were relevant and reported on chiropractic utilization (245 studies), reason for attending chiropractic care (85 studies), patient demographics (130 studies), and assessment and treatment provided (34 studies). Globally, the median 12-month utilization of chiropractic services was 9.1% (interquartile range (IQR): 6.7%-13.1%) and remained stable between 1980 and 2015. Most patients consulting chiropractors were female (57.0%, IQR: 53.2%-60.0%) with a median age of 43.4 years (IQR: 39.6-48.0), and were employed (median: 77.3%, IQR: 70.3%-85.0%). The most common reported reasons for people attending chiropractic care were (median) low back pain (49.7%, IQR: 43.0%-60.2%), neck pain (22.5%, IQR: 16.3%-24.5%), and extremity problems (10.0%, IQR: 4.3%-22.0%). The most common treatment provided by chiropractors included (median) spinal manipulation (79.3%, IQR: 55.4%-91.3%), soft-tissue therapy (35.1%, IQR: 16.5%-52.0%), and formal patient education (31.3%, IQR: 22.6%-65.0%).

**Conclusions:**

This comprehensive overview on the world-wide state of the chiropractic profession documented trends in the literature over the last four decades. The findings support the diverse nature of chiropractic practice, although common trends emerged.

**Electronic supplementary material:**

The online version of this article (10.1186/s12998-017-0165-8) contains supplementary material, which is available to authorized users.

## Background

Chiropractors practice in over 100 countries, in which 90 have established national chiropractic associations [1]. Chiropractic has become one of the most commonly used complementary and alternative medicine (CAM) therapies in the United States and Europe [2, 3]. Chiropractors provide a substantial portion of care for patients with many health conditions including low back and neck pain [4, 5]. They are also a major stakeholder in the health care expenditures of the United States and Denmark [6–8]; for example, in the United States in 2015, chiropractors provided 18.6 million clinical services under Medicare [9] and overall spending for chiropractic services was estimated at USD $12.5 billion [10].

Very few knowledge syntheses have summarized the results of studies describing the profile of chiropractic services and patients who seek their care [11–13]. Available reviews have only reported partial descriptions of chiropractic services and patients, utilization rates [14, 15] or reasons for attending chiropractic care [16]. Stakeholders of the profession would benefit from a current synthesis of research that comprehensively describes the profile of chiropractic practice and services provided to help inform priorities in education, research and workforce development. These priorities can aim to address the most common presenting conditions, facilitate workforce planning, establish professional norms, and guide curriculum design, quality improvement and guideline initiatives.

The objective of this scoping review was to document the current state of knowledge on the: 1) utilization of chiropractic services; 2) reasons for attending chiropractic care; 3) demographic and health profiles of chiropractic patients; and 4) types of chiropractic assessment and treatment provided worldwide.

## Methods

We used scoping review methodology to collect and organize relevant information to address our broad research question and provide a comprehensive examination of the existing body of literature [17]. We employed rigorous methods based on the recommended framework for scoping reviews by Arksey and O’Malley and Levac et al. [18, 19].

### STEP 1: Identifying the research question

Our scoping review was guided by the following broad research question: *What is known about the utilization rate of chiropractic services, reasons for seeking care, patient profiles, and assessment and treatment provided?*

### STEP 2: Identifying relevant studies

We searched MEDLINE, CINAHL, and the Index of Chiropractic Literature (ICL) from database inception to January 14, 2016 using a combination of keywords and subject headings (MeSH or ChiroSH terms) relevant to four themes; utilization rate of chiropractic services, reasons for seeking care, patient profiles, and assessment and treatment provided. These themes were selected by the author team a priori with each representing an essential element of chiropractic practice. We first developed the search strategy in MEDLINE and subsequently adapted it to the other databases (Additional file 1: Appendix A). We used the Preferred Reporting Items for Systematic Reviews and Meta-analyses (PRISMA)[20] flow chart to track the number of studies at each stage of the review.

### STEP 3: Study selection

#### Inclusion and Exclusion Criteria

We included studies: 1) published in the peer-reviewed literature in English or French; 2) were case series, descriptive, cross-sectional, or cohort studies; 3) described patients who received chiropractic services; and 4) reported at least one of the following: utilization rates of chiropractic services; reasons for attending chiropractic care; profiles of chiropractic patients; and types of chiropractic assessment and treatment provided. Studies were excluded if the methodology was not reported, if they were cadaveric or animal studies, or if they solely assessed profiles of chiropractors and did not report patient data, or clinical outcomes related to chiropractic care.

#### Screening and Agreement

Pairs of independent review authors screened the search results in two phases. All studies were screened using titles and abstracts during the first phase (phase I) to identify relevant, possibly relevant, and irrelevant citations. In the second phase of screening (phase II), possibly relevant articles were screened in full text to determine eligibility. For both phase I and II, a third review author was available to resolve discrepancies when forming consensus.

### STEP 4: Data Charting

We extracted the following data from relevant studies (when available): 1) description of the study (study design, number of patients, country of origin, and study population); 2) description of the patient population (demographic information including age, sex, and occupation); 3) reasons for attending chiropractic services (clinical condition); 4) utilization rate of chiropractic services; 5) diagnosis or assessment procedures used by chiropractors; and 6) chiropractic treatment provided. One review author (PB) extracted the data, which were checked by a second review author to minimize error.

### STEP 5: Collating, summarizing, and reporting the results

#### Analyzing the Data

We descriptively summarized the charted data to include the following:

1. ***Descriptive numerical analysis:*** The nature and distribution of the studies were analyzed with regards to the total number of studies, year of publication, study design, country where studies were conducted, and study population.
2. ***Summary of included study findings:*** We analysed the studies by categorising them into four themes: utilization rates; reasons for attending chiropractic care; profiles of chiropractic patients; and, types of chiropractic assessment and treatment provided. Across all studies within each theme, we identified the median rate and interquartile range (e.g., chiropractic utilization rate), percentage of patients, stratified by study population or condition.
3. ***Implication of the results:*** We report the review findings according to the four themes to ensure that the results would have practical implications for future clinical chiropractic practice, research, and policy.

## Results

### Descriptive numerical analysis

**Fig. 1:**
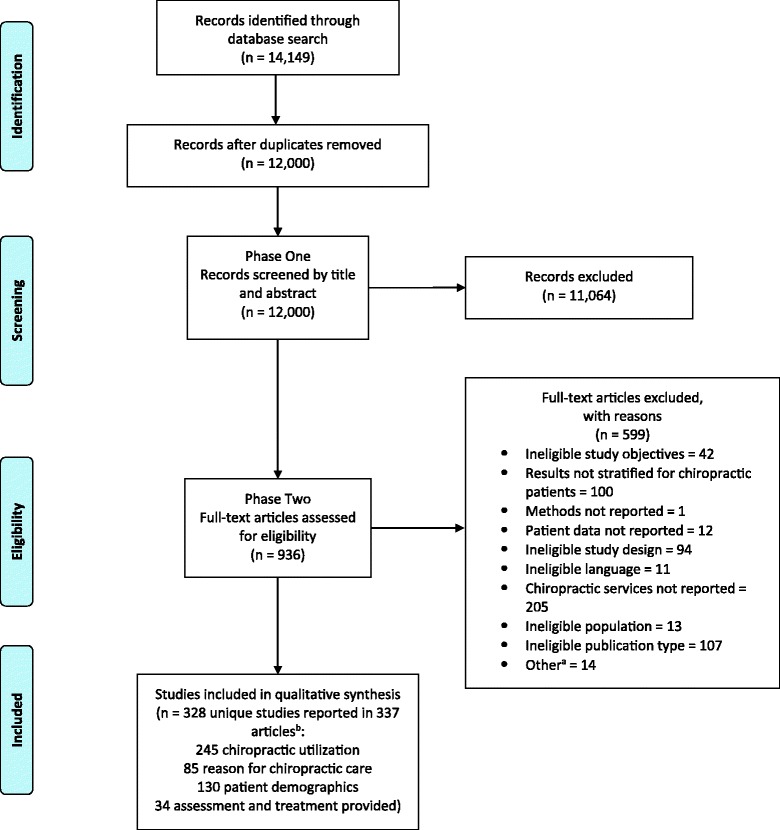
Preferred Reporting Items for Systematic Reviews and Meta-analyses (PRISMA) flow diagram of the scoping review. ^a^Other reasons to exclude articles included issues with article reliability, responsiveness, and interpretability within the context of how the four themes were presented in our review. ^b^Some articles reported more than one topic

The search conducted on January 14^th^, 2016 yielded 14,149 publications, of which 12,000 were screened after removal of duplicates (Fig. 1). Phase I screening excluded 11,064 studies and a further 599 studies were excluded following phase II full text screening. A total of 337 articles (reported on 328 studies) were deemed relevant and were included in this review [11, 21–358]. Additional file 2: Appendix B summarizes the key aspects of each study into the four primary themes. Of the 328 relevant studies, 245 (74.7%) reported utilization rates of chiropractic services, 85 (25.9%) reported reasons for attending chiropractic care, 130 (39.6%) reported patient demographic information, 17 (5.2%) described assessment procedures provided, and 34 (11.0%) described types of chiropractic treatments provided.

**Fig. 2:**
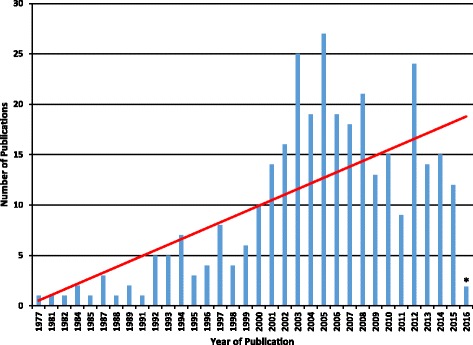
Studies by year of publication showing an increasing trend in the number of relevant publications. *using literature search of MEDLINE, CINAHL, and Index to Chiropractic Literature from database inception to January 14, 2016

We observed an increasing trend in the number of studies published between 1977 and January 2016, with 189 of the 328 studies (57.6%) published between 2005 and 2016 (Fig. 2). Most studies (291 of 328) were cross-sectional, 22 were retrospective cohort, 13 were prospective cohort, one was a chart review, and one was a descriptive study. The studies were most commonly conducted in the United States (*n*=183), Canada (*n*=47), Australia (*n*=41), United Kingdom (*n*=17), and Denmark (*n*=13).

### Review findings

#### Utilization rate of chiropractic services

**Fig. 3:**
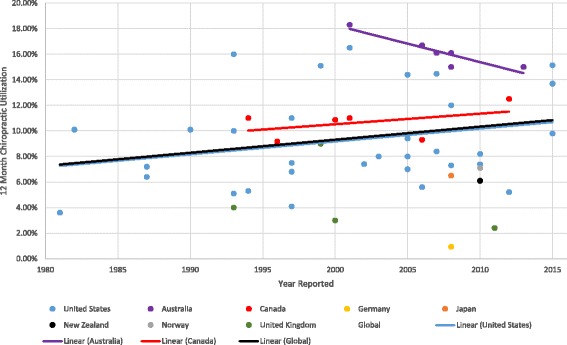
12-month chiropractic utilization between 1980 and 2015

The utilization rate of chiropractic services at the regional or national levels over a 12-month period was reported in 52 studies (Fig. 3). The reported use of chiropractic services generally decreased over time in in Australia from 18.0% to 14.5% and increased over time in Canada and the United States from 10% to 11.7% and from 7.2% to 10.7% respectively. However, no clear worldwide trend of either increased or decreased chiropractic use was observed between 1980 and 2015.

Globally across all 52 studies at the regional/national level that reported utilization, the median 12-month use of chiropractic services was 9.1% (IQR: 6.7%-13.1%) and lifetime utilization was 22.2% (IQR: 12.8%-40.0%) (Table 1). Active military members and veterans used chiropractic services at a similar rate to the general population. Women, patients with chronic pain, and those with back pain (13.0%, 16.1%, and 31.0% respectively), utilized chiropractic services more often than the general population, whereas the pediatric (≤18 years) and older population (≥55 years) used chiropractic services less than the general population. In addition, 40 studies reported chiropractic utilization among unique populations related to specific age groups or health conditions, including psychiatric conditions, multiple sclerosis, stroke, HIV, diabetes, and gastrointestinal conditions. Twelve-month utilisation rates among these populations varied from 0% to 29%

**Table 1:** Utilization of chiropractic services (245 studies)

Study population	Chiropractic utilization rate (median, IQR)	Number of reporting studies
Population at regional/national level		
12-month utilization	9.1% (6.7-13.1)	52
Lifetime utilization^a^	22.2% (12.8-40.0)	19
Women		
12-month utilization	13.0% (8.5-17.3)	12
Lifetime utilization^a^	24.9% (18.2-31.6)	2
Older adults^b^		
12-month utilization	8.4% (5.9-11.8)	14
Lifetime utilization^a^	20.3% (14.6-26.0)	5
Active military and veterans		
12-month utilization	9.1% (7.9-10.5)	5
Pediatric population^c^		
12-month utilization	8.1% (3.8-20.0)	15
Lifetime utilization^a^	11.1% (4.0-21.6)	18
Individuals with cancer		
12-month utilization	13.0% (11.6-13.0)	5
Post-diagnosis utilization^d^	9.1% (8.0-9.2)	5
Lifetime utilization^a^	14.7% (6.0-36.0)	14
Individuals with chronic pain		
12-month utilization	16.1% (11.3-24.2)	6
Lifetime utilization^a^	23.6% (22.6-35.0)	5
Individuals with back pain		
12-month utilization	31.0% (26.2-37.3)	17
Lifetime utilization^a^	31.9% (24.6-46.0)	6

*IQR* interquartile range

^a^Lifetime utilization = use in more than past 12 months

^b^Older adults: ≥55 years of age

^c^Pediatric population: ≤ 18 years of age

^d^Post-diagnosis utilization = lifetime use of chiropractic services following being diagnosed with cancer

#### Reasons for attending chiropractic care

In the general population, musculoskeletal conditions were the predominant reason for attending chiropractic care. The most common reasons were low back or back conditions (median of 49.7% patients (IQR: 43.0%-60.2%)) and neck conditions (22.5% (IQR: 16.3%-24.5%)). In the paediatric population (aged ≤18 years), the most common reason for attending chiropractic care was musculoskeletal conditions, with a median of 44.0% (IQR: 34.7%-57.0%) (Table 2). Only 3.1% (IQR: 1.6%-6.1%) of the general population sought chiropractic care for visceral/non-musculoskeletal conditions (Table 3).

**Table 2:** Reasons for attending chiropractic care reported by the pediatric population^a^ (7 studies)

Reason for attending chiropractic care	Percentage of patients (median, IQR)	Number of reporting studies*
Musculoskeletal conditions	44.0% (34.7-57.0)	7
Excessive crying	19.8% (10.0-29.6)	2
Neurologic conditions	17.9% (12.0-23.7)	2
Gastrointestinal conditions	17.5% (10.7-40.3)	4
Ear, nose, and throat conditions	8.3% (3.0-10.0)	3
Infection	7.0% (4.9-8.0)	3
Headache	6.8% (6.5-7.0)	2
Asthma	5.3% (2.0-8.5)	2
Stomach conditions	5.0% (2.0-8.0)	2
Other	5.0% (0.5-6.0)	3

*IQR* interquartile range

*some studies reported more than one reason for attending care

^a^Pediatric population: ≤ 18 years of age

**Table 3:** The ten most frequently reported reasons for attending chiropractic care and the percentage of patients who sought chiropractic care for each condition (78 studies)

Reason for attending care	Percentage of patients (median, IQR)	Number of reporting studies*
Low back/back pain	49.7% (43.0-60.2)	50
Neck pain	22.5% (16.3-24.5)	36
Extremity problem	10.0% (4.3-22.0)	32
Wellness/maintenance	7.5% (3.0-14.0)	17
Hip pain	7.0% (0.8-10.8)	6
Headache	5.5% (4.0-9.3)	30
Unspecified/miscellaneous/other	5.0% (2.5-8.0)	23
Shoulder/arm pain	5.0% (3.8-7.2)	12
Visceral/non-musculoskeletal	3.1% (1.6-6.1)	15
Knee pain	2.9% (2.6-5.0)	5

*IQR* interquartile range

*some studies reported more than one reason for attending care

#### Profiles of chiropractic patients

People who sought chiropractic care were more likely to be female (median 57.0%, IQR: 53.2%-60.0%) with a median age of 43.4 years (IQR: 39.6-48.0) (Table 4). In general, 77.3% (IQR: 70.3%-85.0%) of the chiropractic patient population were employed, and a smaller proportion were either retired (median 10.7% (IQR: 7.5%-16.0%)), unemployed (median 9.0% (IQR: 2.4%-16.5%)), or students (median 4.0% (IQR: 2.6%-10.6%)). People with disabilities constituted only 1.4% (median IQR: 1.4%-3.0%) of chiropractic patients.

**Table 4:** Characteristics of chiropractic patients (130 studies)

Sex		
Patient population	Percent female (median, IQR)	Number of reporting studies
Regional/national population	57.0% (53.2-60.0)	45
Older adults^a^	60.1% (56.0-66.9)	7
Serving military and veterans	12.0% (6.0-12.0)	3
Pediatric population^b^	47.0% (43.0-50.0)	7
Individuals with cancer	-	0
Individuals with chronic pain	-	0
Individuals with back problems	49.0% (45.0-52.0)	12
Age		
Patient population	Age in years (median, IQR)	Number of reporting studies
Regional/national	43.4 (39.6-48.0)	18
Older adults^a^	76.0 (72.9-79.2)	3
Serving military and veterans	55.0 (54.8-65.0)	3
Pediatric population^b^	7.6 (7.5-10.1)	5
Individuals with cancer	-	0
Individuals with chronic pain	-	0
Individuals with back problems	43.0 (41.5-43.8)	8
Employment (general population)		
Employment type	Percent (median, IQR)	Number of reporting studies
Employed (general)	77.3% (70.3-85.0)	11
Full-time employment	55.8% (44.5-59.0)	10
Self-employed	14.1% (4.6-14.9)	7
Part-time employment	11.0% (8.6-16.9)	10
Home duties	10.9% (6.4-13.8)	14
Retired	10.7% (7.5-16.0)	15
Student	4.0% (2.6-10.6)	15
Unemployed	9.0% (2.4-16.5)	22
Unable to work/disabled	1.4% (1.4-3.0)	5

*IQR* interquartile range

^a^Older adults: ≥55 years of age

^b^Pediatric population: ≤ 18 years of age

#### Types of chiropractic services (assessment and treatment)

For diagnosis or assessment, static palpation was the most commonly used assessment procedure, reportedly used by 89.3% (IQR: 88.4%-95.0%) of chiropractors (Table 5). Other commonly used assessment procedures included motion palpation (86.5%, IQR: 78.0%-88.5%), spinal examination (79.5%, IQR: 79.0%-80.0%), orthopedic tests (71.8%, IQR: 35.5%-85.2%), and neurological examination (64.6%, IQR: 54.0%-77.0%). A third of chiropractors reported using x-rays as an assessment tool (median 35.0% (IQR: 26.5%-59.0%)), while magnetic resonance imaging and computerized tomography were used by only 1.3% (IQR: 1.0%-2.2%) and 1.9% (IQR: 0.3%-1.9%) of chiropractors, respectively.

**Table 5:** Use of diagnostic assessment procedures by chiropractors in patient care (17 studies)

Assessment procedure	Proportion of use (median, IQR)	Number of reporting studies
Static palpation	89.3% (88.4-95.0)	4
Motion palpation	86.5% (78.0-88.5)	4
Spinal examination	79.5% (79.0-80.0)	1^a^
Orthopedic examination	71.8% (35.5-85.2)	6
Neurological examination	64.6% (54.0-77.0)	6
Soft tissue examination	56.0%^b^	1
Postural analysis	48.0% (38.0-70.8)	4
General physical examination	35.0% (29.0-100.0)	3
X-rays	35.0% (26.5-59.0)	12
Complete history	31.0% (23.0-71.3)	4
Vital signs	26.5% (18.0-40.2)	5
Extremity examination	20.5% (16.0-25.0)	1^a^
Examination of lungs	15.8% ^b^	1
Examination of abdomen	13.2% (5.0-21.3)	2
Examination of mouth	13.0% ^b^	1
Examination of heart	10.7% (5.0-16.4)	2
Laboratory tests/referral	6.8% (5.0-14.2)	6
Magnetic resonance imaging	1.3% (1.0-2.2)	3
Computerized tomography	1.9% (0.3-1.9)	1^a^

*IQR* interquartile range

^a^The study consisted of more than one sample and reported the proportion of use for a given assessment procedure within each sample. IQR was calculated using the proportion of use reported in each sample

^b^No IQR reported

Nearly four out of every five people (79.3%, IQR: 55.4%-91.3%) who sought chiropractic care received spinal manipulation, which was the most common treatment provided by chiropractors (Table 6). Other common treatments provided by chiropractors included soft-tissue therapy (35.1%, IQR: 16.5%-52.0%), and formal patient education (31.3%, IQR: 22.6%-65.6%).

**Table 6:** Chiropractic treatment provided and proportion of use (34 studies)

Chiropractic treatment provided		
Chiropractic treatment	Percentage of treatment provided (median, IQR)	Number of reporting studies
Spinal manipulation^a^	79.3% (55.4-91.3)	22
Soft-tissue therapy^b^	35.1% (16.5-52.0)	18
Formal patient education	31.3% (22.6-65.6)	15
Nutritional supplements	30.9% (10.8-63.0)	11
Exercise instruction/prescription	26.0% (9.0-68.1)	14
Cold/ice	26.0% (9.0-74.0)	7
Heat	21.8% (12.0-49.0)	5
Mobilization/Manual traction^c^	17.2% (12.4-32.0)	8
Orthopedic supports	13.0% (3.0-23.0)	2
Electrical stimulation	12.7% (7.9-31.0)	9
Ultrasound	12.5% (6.7-27.1)	7
Acupuncture	2.4% (6.0-1.8)	4
Chiropractic system used		
Chiropractic system	Proportion of use (median, IQR)	Number of reporting studies
Diversified	65.5% (57.1-83.0)	13
Nimmo-Tonus	28.8% (15.6-40.0)	4
Activator	23.0% (14.0-38.0)	12
Gonstead	14.0% (8.0-21.0)	12
Cox	10.5% (4.0-27.0)	5
HIO	10.0% (1.9-16.6)	3
Applied kinesiology	10.0% (3.0-19.0)	6
Thompson	7.0% (2.8-14.0)	6
Cranial/SOT	6.6% (5.8-14.0)	8

*IQR* interquartile range, *HIO* hole in one technique, *SOT* sacro-occipital technique

^a^Spinal manipulation included adjustment with mechanical assistance such as hand-held instruments (Activator), toggle boards, and drop pieces

^b^Soft-tissue therapy included the use of massage techniques, trigger-point release, ischemic compression, and other manual therapies typically used with the intent to treat connective tissue, muscles, and other soft tissue structures

^c^Mobilization/Manual traction included any manual therapy typically directed at the joint complex with varying speeds and amplitudes with the intent to restore optimal motion, function, and to reduce pain

## Discussion

This scoping review of the chiropractic literature, the most comprehensive that we are aware of, identified 328 discrete studies from 337 articles related to the utilization rate of chiropractic services, reasons for attending chiropractic care, demographic and health profiles of chiropractic patients, and types of chiropractic assessment and treatment provided. The majority of these studies were published in the last decade (between 2005 and 2015), used a cross-sectional design, and were conducted in the United States, Canada, and Australia.

Our review’s finding of a regional/national 12-month utilization rate of chiropractic services (median of 9.1% across studies) was comparable to that of previous systematic reviews by Lawrence and Meeker (2007) and Cooper et al. (2013), who reported chiropractic utilization rates ranging between 6% and 12% [14, 15]. However, the variability among reported utilization rates between studies makes interpretation of our findings difficult. Relatively high use of chiropractic services among middle-aged women has also been noted by several prior investigations [359–361]. Patients who had chronic pain and back pain reported higher chiropractic utilization (16.1% and 31.0% respectively), when compared to that of the general population. Higher reported chiropractic utilization was also found by two previous systematic reviews that assessed chiropractic utilization of those who experienced chronic pain and back pain [362, 363]. As most studies assessed chiropractic utilization in the United States, Canada, or Australia, further research is needed to assess utilization in other countries.

Reasons for attending chiropractic care varied between studies. Hestbaek and Stochkendahl (2010) conducted a systematic review of the literature pertaining to the reasons why patients aged between 2-18 years attended chiropractic care. The authors concluded that musculoskeletal conditions, specifically spinal pain, were the main reasons for pediatric patients attending a chiropractor [16]. Our review findings show that musculoskeletal conditions, specifically those of the back and neck regions, are the main reasons for patients of all ages to consult chiropractors.

Our review found that chiropractors offer many types of treatments to their patients, but spinal manipulation was the most common treatment provided. The United States National Center for Health Statistics found that manipulation was the most commonly used provider-based CAM therapy among adults and children [364]. While spinal manipulative therapy (SMT) is an effective strategy for managing back pain [365], recent clinical practice guidelines [366–368] recommend clinicians use multimodal care including patient education and advice, manual therapy (including SMT and mobilisation), supervised and home exercise to increase the likelihood of favourable health outcomes. Our findings suggest that many chiropractors do provide multimodal care, consistent with clinical practice guidelines. Such multimodal care includes SMT, formal patient education, soft-tissue therapy, mechanically-assisted manipulative therapy, nutritional supplements, exercise instruction, ice, heat, mobilization/manual traction, orthopedic supports, electrical stimulation, therapeutic ultrasound, and acupuncture. Therefore, chiropractic care should not be considered consisting exclusively of spinal manipulation.

Stakeholders of the chiropractic profession need to be aware of the current state of knowledge to help inform their decision-making processes, promote evidence-based research informed practice, and to best serve those who seek chiropractic care. This review presents a comprehensive overview on the state of the chiropractic profession and has documented trends in the literature over time.

### Strengths and Limitations

A strength of our study was the systematic process used to collect and summarize the available evidence from a large and diverse body of literature. A scoping review is the most appropriate method to collect and organize diverse information and to develop a picture of the existing evidence base [17]. Rigorous scoping review methodology can be used to bring theory to practice, and to reduce bias when informing stakeholders [369, 370]. We employed rigorous methods based on the recommended framework for scoping reviews by Arksey and O’Malley and Levac et al. [18, 19]***.*** Moreover, we conducted systematic searches of the literature using multiple databases, a combination of key words and subject headings, and without date restrictions. Study selection was based on a set of clear inclusion and exclusion criteria to ensure that consensus between review authors was transparent and reproducible. Finally, we ensured that the charted data from each relevant study was accurate through a second check of the data.

Our scoping review has limitations. While a robust methodology was used, scoping reviews are a relatively new approach for which there is not yet a universal study definition or established methods [371]. However, the framework for scoping reviews published by Arksey and O’Malley in 2005 and expanded by Levac et al. in 2010 is being increasingly used across many disciplines and fields of study [372]. Despite conducting independent screening of systematic searches of multiple databases to identify relevant studies, our review did not review reference lists of included studies or employ hand searching nor were searches conducted in EmBase or AMED. This may have resulted in relevant studies being missed. Studies were excluded if not published in English or French, which may have resulted in missing relevant studies. However, it has been noted that chiropractic journals publish in English, which is recognized as the standard language of science, thereby reducing this risk [373]. Finally, the lack of critical appraisal of the methodology of the included studies may limit this review’s ability to report accurate results due to the inclusion of lower quality research. The implied systematic bias of these individual lower quality studies, and lower overall confidence in our results when summarizing findings, may have resulted in larger/faulty associations and imprecision around the estimates. However, several authors of scoping reviews have argued that by not addressing the issue of quality appraisal, scoping reviews are able to deal with a greater range of study designs and methodologies [374, 375]. The emphasis of a scoping review is on comprehensive coverage rather than on a particular standard of evidence [374, 375].

## Conclusion

We employed rigorous scoping review methodology to document the current state of knowledge on the utilization rate of chiropractic services, demographic and health profiles of people receiving chiropractic care, and the assessment undertaken and treatment provided by chiropractors. Our findings reaffirm that the profile of chiropractic services and patients are diverse, yet common trends emerged. Across different countries and regions, the average 12-month utilization rate of chiropractic services was 9.1% with little change between 1980 and 2015. Musculoskeletal conditions, such as back and neck pain, were the predominant reason for people attending chiropractic care. Typically, chiropractic patients were female, aged 43.4 years, and employed. Four out of five patients who consulted a chiropractor received spinal manipulation; however, chiropractors also commonly provided other treatments including soft-tissue therapy and formal patient education.

## Additional files

Additional file 1: Appendix A. The chiropractic profession a scoping review (DOCX 13 kb)

Additional file 2: Appendix B. The chiropractic profession a scoping review (DOCX 472 kb)
