# Supplementary material for: The chiropractic profession: a scoping review of utilization rates, reasons for seeking care, patient profiles, and care provided
Source: Chiropr Man Therap. 2017 Nov 22;25:35. doi: 10.1186/s12998-017-0165-8 (PMC5698931; doi:10.1186/s12998-017-0165-8)
Supplement: Supplementary file 1 — The chiropractic profession a scoping review (DOCX 13 kb) [file 12998_2017_165_MOESM1_ESM.docx]

**APPENDIX A:**

**MEDLINE (EBSCO) Search Strategy**

1. MH Chiropractic
2. TI chiropract* or AB chiropract*
3. MH Manipulation, Chiropractic
4. MH Complementary Therapies
5. MH Manipulation, Spinal
6. alternative n2 (care or health or medicine* or practitioner*)
7. TI complementary n2 (care or health or medicine* or practitioner* or therap*) or AB complementary n2 (care or health or medicine* or practitioner* or therap*)
8. unconventional n2 (health or practitioner* or therap* or medicine*)
9. non-conventional n2 (health or practitioner* or therap* or medicine*)
10. use* n2 (cam or frequen* or complement* or alternative* or service* or trend* or chiropract* or pattern* or non-conventional or characteristic* or profile* or reason* or visit*)
11. usage* n2 (cam or frequen* or complement* or alternative* or service* or trend* or chiropract* or pattern* or non-conventional or cost* or prevalence)
12. under-use* n2 (cam or frequen* or complement* or alternative* or service* or trend* or chiropract* or pattern* or non-conventional or cost* or prevalence or visit*)
13. TI (utiliz* or utilis*) or AB (utiliz* or utilis*)
14. TI treatment n2 (seek* or sought) or AB treatment n2 (seek* or sought)
15. patient* n2 (characteristic* or complaint* or pattern* or profile* or reason* or visit*)
16. cam n1 service*
17. TI (socio-economic* or socioeconomic* or demograph* or sociodemograph* or socio-demograph*) or AB (socio-economic* or socioeconomic* or demograph* or sociodemograph* or socio-demograph*)
18. MH Socioeconomic Factors
19. chiropractic patient*
20. chiropract* n1 visit*
21. (cam n1 visit*) or (prevalen* n1 visit*)
22. (visit n2 length*) or (total n2 visit*) or (clinic n2 visit*)
23. MH Office Visits
24. MH Patient Acceptance of Health
25. MH Questionnaires
26. MH Health Services/ec/sn/td/ut
27. MH Health Care Surveys
28. visit-based
29. perceive* n2 value
30. MH Professional Practice/sn
31. MH Physician’s Practice Patterns
32. MH Delivery of Health Care
33. MH Health Knowledge, Attitudes, Practice
34. TI expenditur* or AB expenditur*
35. “spatial accessibility”
36. TI (care n2 seek*) or care-seek* or AB (care n2 seek*) or care-seek*
37. 1-9/ OR
38. 10-36/OR
39. 37 AND 38
40. 39 NOT cadaver*
41. 40 NOT (animal* NOT human)
