# Supplementary material for: The chiropractic profession: a scoping review of utilization rates, reasons for seeking care, patient profiles, and care provided
Source: Chiropr Man Therap. 2017 Nov 22;25:35. doi: 10.1186/s12998-017-0165-8 (PMC5698931; doi:10.1186/s12998-017-0165-8)
Supplement: Supplementary file 2 — The chiropractic profession a scoping review (DOCX 472 kb) [file 12998_2017_165_MOESM2_ESM.docx]

**APPENDIX B:**

**Table 1: Utilization of chiropractic services reported in relevant studies (245 studies reported in 251 articles)**

| First author | Year | Study design | Number of patients | Country | Patient population | Utilization rate of chiropractic services |
| --- | --- | --- | --- | --- | --- | --- |
| Adams[22] | 2014 | cross-sectional survey | 214 | Canada | pediatric gastroenterology | 27.3% current use |
| Adams[21] | 2014 | cross-sectional survey | 176 | Canada | pediatric cardiology | 20% current use |
| Adams[23]*  And  Adams[24] | 2011 | cross-sectional survey | 1427 | Australia | National, women (aged 45-50) | 43.0%, 12-month use |
| Adams[25] | 2011 | cross-sectional survey | 8795 | Australia | National, women | 14% pregnant 12-month use, 15% non-pregnant 12-month use |
| Aickin[26] | 2013 | retrospective cohort | 8148 | United States | CAM users with back pain | 54% lifetime use |
| Al-Windi[30] | 2004 | cross-sectional survey | 1433 | Sweden | Adults aged 16 years and above | 14.2% lifetime use |
| Astin[31] | 2000 | cross-sectional survey | 728 | United States | Elderly, Blue Shield Medicare members | 20.3% 12-month use |
| Ayers[32] | 2010 | cross-sectional survey | 30,923 | United States | National, adults | 7.38% 12-month use |
| Berecki-Gisolf[34] | 2013 | retrospective cohort | 51263 | Australia | Whiplash patients claiming Transport Accident Commission compensation | 5% lifetime use |
| Boon[36] | 2000 | cross-sectional survey | 422 | Canada | Breast Cancer, women adults | 29.2% lifetime use |
| Broom[40] | 2012 | cross-sectional survey | 9820 | Australia | National, women (aged 45-50 years) | 33% with back pain, 8% without back pain |
| Broom[41] | 2012 | cross-sectional survey | 10492 | Australia | National, women (aged 45-50 years) | 16.1% with back pain, 7.9% without back pain |
| Brown[43] | 2013 | Cross-sectional | 757 | Australia | General public | 39.9% lifetime use |
| Brunelli[44] | 2003 | Cross-sectional | 180 | United States | Patients with peripheral neuropathy from outpatient clinics | 8.9% 5 year use |
| Carey[50]*  And  Carey[51] | 1995 | cross-sectional survey | 4437 | United States | North Carolina, general population with low back pain | 24.6% lifetime use |
| Carey[48] | 1995 | prospective cohort | 1633 | United States | North Carolina patients with acute low back pain | 18.9% urban 24 week use, 18.1% rural 24 week use |
| Carey[49] | 1999 | prospective cohort | 921 | United States | North Carolina patients with acute low back pain that resolved within 3 months | 38.34% 6-month use for recurrent low back pain |
| Chao[52] | 2008 | cross-sectional survey | 10759 | United States | National, general population | 19.9% 12-month use |
| Cheung[54] | 2007 | cross-sectional survey | 1200 | United States | Minnesota, adults >=65 years | 17.8% 12-month use |
| Cleary[55] | 1982 | cross-sectional survey | 1026 | United States | Wisconsin, adults 18+ | 10.1% 12-month use |
| Conboy[56] | 2005 | cross-sectional survey | 2055 | United States | National | 37% lifetime use |
| Cote[57] | 2001 | cross-sectional survey | 907 | Canada | neck pain and/or low back pain | 12.1% back pain use |
| Coulter[60] | 1996 | Descriptive; secondary analysis of RCT | 414 | United States | US, adults >=65 | 5.65% 4-month use |
| Davis[61] | 2010 | retrospective cohort | 1871 | United States | 789 adult chiropractic patients in 1997, 1082 Sampling frame ranged from 22,953 (1998) to 32,737 (2003); response rate ranged from 58X3% to 66X7%; adult reported seeing a chiropractor in previous 6 months | 7.5% 12-month use in 1997, 5.6% 12-month use in 2006 |
| Deyo[62] | 1987 | cross-sectional survey | 10404 | United States | National, adults 25+ with low-back pain | 30.8% lifetime use |
| Dimmock[63] | 1996 | cross-sectional survey | 40 | United Kingdom | fibromyalgia | 8% lifetime use |
| Doering[64] | 2013 | cross-sectional survey | 132 | Germany | pediatric patients with epilepsy | 4% lifetime use |
| D'Onise[65] | 2012 | cross-sectional survey | 1146 | Australia | Rural Australia, people with chronic disease | 24.2% 12-month use |
| Drivdahl[66] | 1998 | cross-sectional survey | 177 | United States | US, adults 18+ | 64% lifetime use |
| Druss[67] | 2003 | Cross-sectional | One or more visits to a nonphysician clinician in 1987: 21,501, 1997: 22,505; one or more visits to a physician and non-physician clinician in 1987: 6414, 1997: 7703 | United States | General population | 6.4% 12-month use 1987, 4.1% 12-month use 1997 |
| Eaves[70] | 2015 | cross-sectional survey | 64 | United States | CAM patients with chronic low back pain | 23.45 lifetime use |
| Eisenberg[74] | 1993 | cross-sectional survey | 1539 | United States | National, adults | 10% 12-month use |
| Eisenberg[73] | 1997 | cross-sectional survey | 3594 | United States | National, adults 18+ | 10.1% 12-month use 1990, 11.0% 12-month use 1997 |
| Elder[75] | 2015 | cross-sectional survey | 6068 | United States | Oregon and Washington, patients with chronic musculoskeletal pain | 47% 12-month use |
| Elder[76] | 1997 | cross-sectional survey | 113 | United States | Oregon | 42% lifetime use |
| Elkins[77] | 2005 | cross-sectional survey | 82 | United States | Psychiatric inpatients | 4% 12-month use |
| Emslie[79] | 2002 | cross-sectional survey | 773 | United Kingdom | National | 4% 12-month use 1993, 9% 12-month use 1999 |
| Enyinnaya[81] | 2012 | prospective cohort | 12440 | United States | Patients with arthritis | 11.3% 24-month use rural, 6.0% 24-month use non-rural |
| Ernst[82] | 2000 | cross-sectional survey | 1204 | United Kingdom | National, adults | 3% 12-month use |
| Evans[83] | 2008 | cross-sectional survey | 92 | New Zealand | Hospital patients | 51% lifetime use |
| Factor-Litvak[84] | 2001 | cross-sectional survey | 300 | United States | New York City, women | 18.2% lifetime use |
| Fadanelli[85] | 2012 | Cross-sectional | 112 | Italy & Canada | Children with diagnosed rheumatic disease | 5% use Italy, 21% use Canada |
| Fautrel[86] | 2002 | cross-sectional survey | 66,000 | Canada | National survey (noninstitutionalized population) | 59.3% 12-month use arthritis or rheumatism, 75.6% 12-month use chronic back problems, 49.9% 12-month use other chronic conditions |
| Fawcett[87] | 1994 | cross-sectional survey | 16 | United States | multiple sclerosis | 38% lifetime use |
| Featherstone[88] | 2003 | cross-sectional survey | 1174 | United Kingdom | GP patients in Scotland | 10.8% lifetime use, 2.4% 1-month use |
| Feinglass[89] | 2007 | cross-sectional survey | 763 | United States | Arthritis, adults 45+ | 0.7% 12-month use south Chicago, 4.3% 12-month use north Chicago |
| Feldman[90] | 2004 | cross-sectional survey | 118 | Canada | Juvenile idiopathic arthritis | 1.7% 3-month use |
| Feldman[91] | 2004 | cross-sectional survey | 518 | Australia | University students | 13.9% lifetime use |
| Fernandez[92] | 1998 | cross-sectional survey | 583 | Canada | pediatric patients attending oncology centre | 3.25% lifetime use |
| Feuerstein[93] | 2004 | cross-sectional survey | 2,135 | United States | National, patients with nonspecific back pain | 40.48% 12-month use 1987, 30.63% 12-month use 1997 |
| Fitzcharles[94] | 1997 | Cross-sectional | 82 | Canada | Female Patients with fibromyalgia | 12% fibromyalgia use |
| Flaherty[95] | 2001 | cross-sectional survey | 593 | United States & Japan | Geriatric outpatients in US and Japan local hospitals | 9% geriatric use |
| Fleming[96] | 2007 | cross-sectional survey | 908 | United States | Wisconsin primary care patients (18-81yrs) using opioid therapy for chronic pain | 23.6% lifetime use, 17.8% 12-month use |
| Fong[97] | 2002 | cross-sectional survey | 120 | Australia | pediatric patients | 12% lifetime use |
| Fortier[98] | 2014 | cross-sectional survey | 206 | United States | Mothers of pediatric patients undergoing surgery | 62% lifetime use English Caucasian, 32% lifetime use English Hispanic, 12% lifetime use Spanish Hispanic |
| Foster[99] | 2000 | cross-sectional survey | 311 | United States | adults 65+ | 11% 12-month use >65 years aged |
| Fouladbakhsh[100] | 2008 | Cross-sectional | General population with cancer: 2,262; without cancer 28,734 | United States | General population with cancer and without cancer | 7.3% use non-cancer population, 8.2% use cancer population |
| Fouladbakhsh[101] | 2005 | cross-sectional survey | 968 | United States | Oncology patients | 2.8% lifetime use |
| Fox[102] | 2013 | cross-sectional survey | 406 | Ireland | Breast cancer | 0.5% lifetime use |
| Frawley[103] | 2013 | cross-sectional survey | 1835 | Australia | Pregnant women | 16.3% pregnant women use |
| French[105] | 2013 | cross-sectional survey | 7519 | Australia | General medical practice patients who reported they used chiropractic services in last 12 months | 15% 12-month use |
| Frenkel[106] | 2008 | cross-sectional survey | 502 | United States | adults patients | 12.0% 12-month use |
| Friedman[107] | 1997 | cross-sectional survey (interviews) | 161 | United States | pediatric oncology (Hematology) patients vs controls | 1.2% cancer patients use, 1.3% non-cancer patient use |
| Furler[108] | 2003 | cross-sectional survey | 104 | Canada | HIV-infected patients | 19.2% lifetime use |
| Furlow[109] | 2008 | cross-sectional survey | 483 | United States | obstetrics and gynecology | 4.8% lifetime use |
| Gaedeke[110] | 1999 | cross-sectional survey | 485 | United States | College students | 25.8% lifetime use |
| Gaffrey[111] | 2004 | Cross-sectional | 220 | Australia | Women (18+) at least 36 weeks gestation at antenatal clinic | 10.9% use during pregnancy |
| Ganguli[112] | 2004 | cross-sectional survey | 341 | Canada | gastroenterology patients | 21.4% 12-month use |
| Garrow[113] | 2006 | cross-sectional survey | 31099 | United States | Diabetes, adults | 20.8% 12-month use, 19.4% 12-month diabetic use |
| Gaumer[114] | 2006 | cross-sectional survey | 2398 | United States | visited a chiropractor before | 12% lifetime use |
| George[115] | 2011 | prospective cohort | 500 | United States | military primary care patients | 21% 5-year use |
| Gerasimidis[116] | 2008 | cross-sectional survey | 86 | United Kingdom | inflammatory bowel disease | 5% lifetime use |
| Geva[117] | 2005 | cross-sectional survey | 368 | Israel | Cancer patients | 0.54% lifetime use |
| Goertz[119] | 2013 | cross-sectional survey | 16146 | United States | military primary care patients | 5.2% 12-month use |
| Goldman[120] | 2015 | cross-sectional survey | 2467 | United States | General population | 15.14% bridges using use, 9.8% non-bridges using use |
| Goldstein[121]*  And  Goldstein[122] | 2005 | cross-sectional survey (Telephone survey) | 9187 (1844 with cancer) | United States | Oncology patients, adults | 13% 12-month use, 36.0% lifetime use |
| Gore[123] | 2012 | Cross-sectional | 64085 with OA, 47386 with chronic LOW BACK PAIN | United States | Patients (18+) with osteoarthritis and or chronic low back pain in managed healthcare plan during 2008 in community based settings | 34.1% 12-month chronic low back pain use, 10.5% 12-month osteoarthritis use |
| Gore-Felton[124] | 2003 | Cross-sectional | 158 | United States | Patients (18+) with HIV | 8.9% lifetime use |
| Graham[125] | 2013 | cross-sectional survey | 434 | Canada | pediatric oncology (otolaryngology) | 2.0% lifetime use |
| Graham[126] | 2005 | cross-sectional survey | 31,044 | United States | National, adults | 7% 12-month use |
| Gray[127] | 2002 | cross-sectional survey | 4404 (86%response rate) | United States | Chronic disease, 40+ | 8% 12-month use |
| Gray[128] | 2003 | cross-sectional survey | 731 | Canada | Breast Cancer, women adults | 8% post diagnosis use |
| Greenfield[129] | 2002 | cross-sectional survey | 150 | United Kingdom | Students, first year medical | 5.3% lifetime use |
| Grossoehme[130] | 2013 | Cross-sectional | 25 | United States | Parents of children with cystic fibrosis | 5% lifetime use |
| Grzywacz[131] | 2006 | cross-sectional survey | 5827 | United States | National, adults 65 and older | 5.5% 12-month use with anxiety or depression |
| Gulla[132] | 2000 | cross-sectional survey | 139 | United States | Emergency Department Patients | 30% lifetime use |
| Habermann[133] | 2009 | cross-sectional survey | 56 | United States | Lymphoma, survivors 5 to 20 years | 39% lifetime use |
| Hagen[134] | 2003 | cross-sectional survey | 141 | Canada | Pediatric rheumatology patients | 21% lifetime use |
| Hall[135] | 2014 | cross-sectional survey | 315 | United Kingdom | Women during pregnancy | 0.6% use during pregnancy |
| Hamm[136] | 2014 | cross-sectional survey | 54,437 | United States | National, adults | 7.4% 12-month use 2002, 8.39% 12-month use 2007 |
| Hanley[137] | 2006 | cross-sectional survey | 255 | United States | Lower-limb Phantom pain | 4% lifetime use |
| Hann[139] | 2005 | cross-sectional survey | 608 | United States | Breast Cancer, women adults | 10% lifetime use |
| Hann[138] | 2005 | cross-sectional survey | 208 | United States | Breast and Prostate cancer patients | 18% breast cancer use, 7% prostate cancer use |
| Hansen[140] | 1997 | Cohort | 1993 n=31,225; 1994 n=31,800 | United States | Group Health Cooperative (HMO) patients | 5.1% 12-month use 1993, 5.3% 12-month use 1994 |
| Hanssen[141] | 2005 | cross-sectional survey | 27691; Norway=1000; Denmark=16,690; Sweden=1001; Total=18,691 | Norway; Denmark; Sweden | Multi-national; Norway-national; Denmark- national; Sweden | 11% lifetime use Norway, 30% lifetime use Sweden |
| Harding[142] | 2009 | cross-sectional survey | 99 | United Kingdom | Non-elite marathon runners | 11% 12-month use injured runners |
| Harrigan[143] | 2006 | cross-sectional survey | Hawai’i 5000; US not stated | United States | National & Hawaiian, adults over 18 years of age | 30.4% lifetime use Hawai’i, 19.9% lifetime use United States |
| Harrington[144] | 2006 | cross-sectional survey | 77 | United States | Children (2-19 years old) with autistic spectrum disorders | 21.6% lifetime use, 18.9% lifetime use Autism, 27.6% lifetime use Pervasive developmental disorder-not otherwise specific, 13.0% lifetime use Asperger’s Syndrome |
| Hartmann[145] | 2016 | cross-sectional survey | 21 164 | Germany | Children & Adolescents (0-18 years old) with epilepsy who used CAM | 1.2% lifetime use |
| Hawk[151] | 1999 | cross-sectional survey | 1511 | United States | seen a chiropractor for any physical problem within the past year | 15.1% 12-month use |
| Hazra[154] | 2010 | Cross-sectional | 172 | Canada | Ambulatory patients with schizophrenia and related primary psychotic disorders at inner city hospital | 8.7% 12-month use, 20.9% lifetime use |
| Heathcote[155] | 2011 | Cross-sectional | 306 | United States | Adult attendees at free community health clinic | 19.7% lifetime use |
| Helyer[156] | 2006 | cross-sectional survey | 32 | Canada | Breast Cancer, women adults | 6% lifetime use |
| Henderson[157] | 2004 | cross-sectional survey | 551 | United States | Breast Cancer, women adults | 13% 12-month use |
| Hilsden[160] | 2003 | cross-sectional survey | 2847 | Canada | Inflammatory bowel disease | 9.6% 12-month use |
| Hirsh[161] | 2011 | cross-sectional survey | 83 | United States | Adults with cerebral palsy | 31% lifetime use |
| Ho[162] | 2015 | cross-sectional survey | 150 | United States | Hispanic patients | 11% 12-month use US-born, 10% 12-month use non-US-born |
| Ho[163] | 2009 | cross-sectional survey | 92 | United States | Chronic pain | 35% lifetime use |
| Holler[165] | 2002 | cross-sectional survey | 70 | Canada | Pregnant women (presenting with nausea & vomiting) | 18.2% lifetime use |
| Honda[167] | 2005 | cross-sectional survey | 4242 | United States | Adults 25-74 years of age | 9.4% 12-month use no mental nor physical disorder, 10.1% 12-month use with mental disorder, 10.15% 12-month use with physical disorder, 13.6% 12-month use both mental and physical disorder, 10.9% 12-month use total population |
| Hori[168] | 2008 | cross-sectional survey | 496 | Japan | Adults, outpatients in 1 hospital | 6.5% 12-month use |
| Hsiao[169] | 2003 | cross-sectional survey | 2466 | United States | HIV-infected patients | 8.1% post diagnosis use |
| Hsu[170] | 2008 | cross-sectional survey | 206 | Taiwan | Depression, adults (≥50yrs) hospital based | 14.1% 12-month use |
| Hughes[171] | 2006 | cross-sectional survey | 1104 | United States | Pediatric patients | 20% 12-month use |
| Humpel[172] | 2005 | cross-sectional survey | 81 | Australia | Cancer patients, 18 years+ | 9.2% post diagnosis use |
| Hung[174] | 2015 | cross-sectional survey | 269 | United States | Gastrointestinal diseases | 18% 24-month use |
| Hunt[175] | 2010 | cross-sectional survey | 7630 | United Kingdom | National, 16 years aged+ | 2.4% 12-month use, 8.5% lifetime use males, 4.6% lifetime use females |
| Hunter[176] | 2014 | cross-sectional survey | 152 | Australia | Oncology patients | 7.89% post diagnosis use |
| Hurvitz[177] | 2003 | cross-sectional survey | 213 | United States | Cerebral palsy, children | 6.72% cerebral palsy use |
| Hurwitz[179] | 2006 | cross-sectional survey | 1043 | United States and Canada | General population | 11.5% lifetime use United States, 12.8% lifetime use Canada |
| Ivanova[180] | 2011 | retrospective cohort | 211551 | United States | Low back pain in Primary care | 39.2% 12-month use |
| Jacob[181] | 2003 | cross-sectional survey | 555 | Israel | Low back pain patients | 1.3% 12-month use |
| Jacobs[182] | 2004 | cross-sectional survey | 4158 | Canada | Canadian Community Health Survey 2000; Chronic back pain >6mths, adults aged 20 years and older | 32.1% 12-month use |
| Jacobson[184] | 2009 | Cross-sectional | 86,131 | United States | Millennium Cohort Study, active duty military and Reserve/National Guard personnel | 10.5% 12-month use |
| James[185] | 1992 | Cross-sectional | 42 | New Zealand | Pain clinic patients | 14.3% 12-month use |
| Jawahar[189] | 2012 | Cross-sectional | 2679 | United States | Osteoarthritis Initiative (OAI) study participants with radiographic tibiofemoral knee OA confirmed | 5.3% 6-month use females, 3.0% 6-month use males |
| Jean[190] | 2007 | Cross-sectional | 114 | Canada | Parents of patients presenting to paediatric outpatient clinic patients at University affiliated general hospital | 19% 12-month use |
| Jordan[192] | 2000 | Cross-sectional | 937 | United States | Respondents to the 1993/94 National Survey of Self-care and Aging (≥ 65) who reported pain, stiffness or swelling in the previous 12 months | 10% 12-month use |
| Jordan[193] | 2004 | Cross-sectional | X | United Kingdom | Patients (55+) with a clinical diagnosis of knee OA in an urban and a rural practice | 7.7% lifetime use |
| Kaboli[194] | 2001 | Cross-sectional | 480 | United States | Urban and rural community-dwelling adults (≥65) with self-reported physician diagnosis of arthritis or rheumatism | 15% 12-month use, 26.0% lifetime use |
| Kalaaji[196] | 2012 | Cross-sectional | 300 | United States | Adult dermatology patients at academic tertiary facility | 22% use dermatology patients |
| Katz[199] | 2007 | Cross-sectional | 1226 | United States | Adults (≥40) with active knee OA participating in 4 multicenter studies | 1.6% African American use, 3.5% Asian use, 3.7% Caucasian use, 2.9% Hispanic use |
| Keegan[200] | 1996 | Cross-sectional | 213 | United States | Mexican American patients from 2 medical facilities | 19% 12-month use |
| Keenan[201] | 2003 | Cross-sectional | 2602 | United States | Women (≥45) participating in the Conventional, Complementary and Alternative Menopausal Practices Survey (CAMPS) | 2.5% use greater than 3 months |
| Kessler[203] | 2001 | Cross-sectional | 341 | United States | Nationally representative household sample (≥18) with self-defined anxiety attacks or severe depression | 0.5% use Anxiety attacks, 1.0% use depression |
| Kilbourne[204] | 2007 | Cross-sectional | 435 | United States | Continuous Improvement for Veterans in Care-Mood Disorders (CIVIC-MD) study--patients with bipolar disorder | 7.9% use Veterans |
| Kim[205] | 2003 | Cross-sectional | 219 | Korea | Patients (≥18) at rheumatology clinic affiliated with university hospital | 4% use rheumatology patients |
| Kim[206] | 2004 | Cross-sectional | 143 | United States | Korean Americans attending practitioners of alternative medicine | 23.8% use Korean Americans |
| King[207] | 2004 | cross-sectional survey | 60 | USA | Senior center living independently (African American , Appalachian, white) | 15% lifetime use |
| Kinge[208] | 2015 | cross-sectional survey | 25718 | Norway | Norwegian population with chronic musculoskeletal disorders > 6mths | 12% lifetime use |
| Kirby[209] | 2012 | cross-sectional survey | 1310 | Australia | Females with back pain | 37.3% use women with back pain |
| Kleiman[211] | 1981 | cross-sectional survey (1974 Health interview survey) | 116000 | USA | Non-institutionalized pop | 3.6% 12-month use |
| Kozma[213] | 2014 | cross-sectional survey | 85014 | United States | Patients with/without neuropathic neck-back diagnoses and OA diagnoses; | 28.9% use neuropathic diagnosis, 58.8% neuropathic and nociceptive diagnosis, 67.1% use nociceptive diagnosis, 3.1% use osteoarthritis |
| Krauss[214] | 1998 | cross-sectional survey | 401 | USA | Working age individuals with physically disabling conditions | 6.7% use |
| Kronenberg[215] | 2006 | cross-sectional survey | 3068 | USA | Women in four ethnic groups | 8.9% use |
| Lafferty[216] | 2004 | cross-sectional survey | 7915 | USA | Insurance claimants with cancer diagnoses | 11.6% use |
| Lee[220] | 1985 | cross-sectional survey | 3,686,000 | Canada | Canadians with arthritis, rheumatism, or back, limb and joint disorders | 34% use |
| Lewis[222] | 2001 | cross-sectional survey | 416 | Canada | Hamilton-Wentworth medical residents | 20.1% use |
| Lim[223] | 2005 | cross-sectional survey | 503 | Australia | Children | 7% use pediatric patients |
| Lim[224] | 2006 | cross-sectional survey | 113,229 | Canada | Adults | 9.3% 12-month use no low back pain, 26.2% 12-month use with low back pain |
| Lind[225] | 2009 | cross-sectional survey (claims data) | 237,500 | USA | Insurance claimants | 38.1% lifetime use |
| Lind[227] | 2006 | cross-sectional survey | 20,722 | USA | Diabetes Patients | 14.6% use |
| Lind[226] | 2007 | cross-sectional survey | 13,792 | USA | Fibromyalgia patients | 40.9% use |
| Lind[228] | 2005 | cross-sectional survey (claims data) | 104,358 | USA | Insurance claimants with back pain | 46% use back pain |
| Lorea[231] | 2007 | cross-sectional survey | 1,445 | USA | Non-institutionalized Mexican Americans | 2.4% lifetime use |
| Mackenzi[232] | 2003 | cross-sectional survey | 3,789 | USA | Ethnically diverse adults | 8% 12-month use |
| MacLennan[233] | 2006 | cross-sectional survey | 3,015 | Australia | Teens and adults | 16.7% 12-month use |
| Madsen[235] | 2003 | cross-sectional survey | 622 | Denmark | Paediatric patients | 12.2% 1-month use, 74% lifetime use |
| Marchand[237] | 2012 | cross-sectional survey | 19821 | Italy | Pediatric patients (0 to 18 years old) | 8.1% 12-month use general practice, 21.0% 12-month use specialized practice |
| Marrie[238] | 2003 | cross-sectional survey | 20,778 | USA | Multiple Sclerosis patients | 51% lifetime use |
| Marsh[239] | 2009 | cross-sectional survey | 373 | Canada | Osteoarthritis patients | 11.8% current use |
| Martel[240] | 2005 | cross-sectional survey | 92 | Canada | Children with cancer | 7% use |
| Martin[241] | 2012 | cross-sectional survey | 12,036 | USA | Patients with neck and back problems | 26.8% use |
| McEachrane-Gross[243] | 2006 | cross-sectional survey | 264 | USA | Veterans with cancer or chronic pain | 26.4% use Veterans |
| McHardy[244] | 2007 | cross-sectional survey | 1,634 | Australia | Amateur golfers | 40% lifetime use |
| Metcalfe[245] | 2004 | cross-sectional survey | 400,055 | Canada | Canadian adults with chronic disease (asthma, diabetes, epilepsy and migraine) | 11.3% use chronic disease |
| Millar[247] | 2001 | cross-sectional survey | 14150 | Canada | general adult population | 11% 12-month use |
| Millar[246] | 1997 | cross-sectional survey | 17626 | Canada | National population | Age adjusted percentage of population aged 15 and over who consulted chiropractors in Canada, 1994-1995: By region: Canada (11%), Atlantic (3%), Quebec (8%), Ontario (10%), Prairies (17%), British Columbia (17%) |
| Mior[249] | 2008 | cross-sectional survey | not reported | Canada | general adult population | 9.19% 12-month use without back pain 1996, 10.87% 12-month use without back pain 2000. 32% 12-month use back pain 1996, 28% 12-month use back pain 2000 |
| Montalto[250] | 2006 | cross-sectional survey | 848 | United States | general adult population 50 years and over | 60% lifetime use |
| Montgomery[251] | 2011 | cross-sectional survey | 9,289 | United States | Childhood Cancer Survivor | 12.4% 2-year use |
| Murthy[253] | 2014 | cross-sectional survey | 1620 | Australia | Australian women aged 60-65 years with back pain, n=1310 | 37.2% use women |
| Najm[254] | 2003 | cross-sectional survey | 525 | United States | 65+ ethnic people | 16.3% 12-month use |
| National Center for Complementary and Alternative Medicine National Institutes of Health[255] | 2005 | Cross-sectional | 31,000 | United States | General population, adults (18+) | 8% 12-month use |
| Nayak[256] | 2001 | cross-sectional survey | 77 | United States | people with spinal cord injuries and chronic pain | 22.6% lifetime use |
| Nayak[257] | 2003 | cross-sectional survey | 3140 | United States | adults with multiple sclerosis | 25.5% lifetime use |
| Ndetan[258] | 2010 | cross-sectional survey | 24275 | United States | general adult population | 8.2% 12-month use |
| Newton[259] | 2002 | cross-sectional survey | 886 | United States | women aged 45-65 | 31.6% general use, 0.9% use menopausal symptoms |
| Nichols[260] | 2006 | cross-sectional survey | 309 | United States | intercollegiate student athletes attending a Division I NCAA University | 29% 12-month use |
| Opheim[265] | 2012 | cross-sectional survey | 430 | Norway | patients with inflammatory bowel disease | 8% 12-month use |
| Paramore[266] | 1997 | cross-sectional survey | 3450 | United States |  | 6.8% 12-month use |
| Piérard[272] | 2012 | cross-sectional survey | 85,387 | Canada | adult general population | 12.5% 12-month use |
| Pledger[273] | 2010 | cross-sectional survey | 12,529 | New Zealand | 15year and older general population | 6.1% 12-month use |
| Post-White[274] | 2009 | cross-sectional survey | 281 | United States | Children With Cancer and General and Specialty Pediatrics | 25.9% 12-month use |
| Quan[276] | 2008 | cross-sectional survey | 1637 | Canada | Chinese and Caucasian residents of Calgary aged 18 or older | 21.2% 12-month use, 8.4% 12-month use Chinese |
| Reinhard[277] | 2014 | cross-sectional survey | 20563 | United States | Military Operation Enduring Freedom and Operation Iraqi Freedom (OEF/OIF) deployed and non-deployed veterans | 0.5% use OEF/OIF Veterans (deployed), 11.62% use Veterans elsewhere, 0.75% use OEF/OIF Veterans (not deployed), 10.16% use Veterans elsewhere |
| Rivera[278] | 2002 | cross-sectional survey | 547 | United States | people attending hospitals and clinics | 11.8% 12-month use |
| Robinson[280]*  And  Robinson[279] | 2007 | cross-sectional survey | 459 | Australia | general adult population | 50% lifetime use |
| Rosen[281] | 2013 | cross-sectional survey | 1327 | United States | people with thyroid cancer | 29.6% 12-month use |
| Rosenfeld[282] | 1987 | cross-sectional survey | 585 | United States | general adult population | 7.2% 12-month use |
| Ross[283] | 2012 | cross-sectional survey | 218 | United States | men with history of prostate cancer | 50% 12-month use, 78% lifetime use |
| Ryder[285] | 2008 | cross-sectional survey | 95 | United States | African American adults aged 60+ | 4.2% 12-month use |
| Sarris[287] | 2010 | cross-sectional survey | 439 | Australia | National, women aged 40-79 | 7.8% 12-month use aged 40-64 years, 6.8% 12-month use aged ≥65 |
| Sawni-Sikand[288] | 2002 | cross-sectional survey | 1013 | United States | Pediatric patients from 6 pediatric practices | 18% lifetime use |
| Saxe[291] | 2008 | cross-sectional survey | 2527 | United States | Breast cancer survivor patients | 47% lifetime use, 8% use for cancer-related reasons |
| Schwarz[292] | 2008 | cross-sectional survey | 4291 | Germany | Population-based West Pomerania aged 20-79 years | 0.932% 12-month use, 15.7% use among CAM users |
| Scott[293] | 2005 | cross-sectional survey | 127 | United Kingdom | Oncology patients | 9.1% use post diagnosis, 14.7% use prior to diagnosis, 3.1% current use |
| Shah[294] | 2008 | cross-sectional survey | 791 | United States | Stroke patients | 19.4% lifetime use |
| Shakeel[295] | 2009 | cross-sectional survey | 285 | United Kingdom | Otolaryngologic Surgery patients | 9.47% lifetime use, 15.25% lifetime use among CAM users |
| Shakeel[296] | 2010 | cross-sectional survey | 1789 | United Kingdom | Otolaryngologic Surgery patients | 8.83% 12-month use |
| Shekelle[298] | 1991 | retrospective cohort | 5279 | United States | National, under the age of 65 years old | 7.5% 5- year use |
| Sherman[299] | 2004 | cross-sectional survey | 249 | United States | Chronic low back pain patients | 54% lifetime use, 45% lifetime use for low back pain |
| Sherwood[300] | 2008 | cross-sectional survey | 268 | Australia | National population | 15% 12-month use |
| Sinha[302] | 2005 | cross-sectional survey | 75 | Australia | Pediatric patients with ADHD | 10% lifetime use |
| Smith[303] | 2006 | cross-sectional survey | 2985 | Australia | Pediatric patients | 34% 12-month use |
| Soo[304] | 2005 | cross-sectional survey | 105 | Canada | Pediatric neurology patients | 6.6% lifetime use, 15% lifetime use among CAM users |
| Spector[306] | 2012 | cross-sectional survey | 402 | United States | Patients of a dental school clinic | 26.1% 12-month use, 63.4% lifetime use |
| Spigelblatt[307] | 1994 | prospective longitudinal cohort | 1911 | Canada | Pediatric patients | 5.55% lifetime use |
| Stano[308] | 1993 | retrospective longitudinal cohort | 395641 | United States | National, with neuromusculoskeletal ICD-9 codes | 22.9% 2-year use |
| Steinbekk[310] | 2010 | cross-sectional survey | 7888 | Norway | Population-based 13 years and older | 7.1% 12-month use |
| Stevens[314] | 2005 | cross-sectional survey | 118 of 250 surveyed | United States | US residents | 14.4% 12-month use |
| Stude[315] | 2008 | cross-sectional survey | 402 | United States | Golfers attending conference | 47% lifetime use |
| Stump[316] | 2002 | cross-sectional survey | 22 | United States | NFL trainers | 45% lifetime use |
| Sullivan[318] | 2015 | cross-sectional survey | 142 | Australia | cancer patients | 27% lifetime use not related to cancer |
| Thorburn[319] | 1993 | cross-sectional survey | 6722 | United States | National Health Care Quality Survey 2001, adults 18yrs> | 16% 2-year use |
| Torres-Llenza[322] | 2010 | cross-sectional survey | 2027 | Canada | Children with asthma presenting to Asthma Centre in an Urban hospital | 0.8% use, 9% use among CAM users |
| Upchurch[323] | 2005 | cross-sectional survey | 17599 | United States | Women, 18 years and older; National Health Interview Survey 1999 | 8.1% 12-month use |
| Valji[324] | 2013 | cross-sectional survey | 129 | Canada | Pediatric oncology patients from two Urban hospitals | 10.1% lifetime use, 3.8% current use |
| Votova[325] | 2007 | cross-sectional survey | 4401 | Canada | National Population Health Survey 1994; older adults, aged 50 years and older | 11.9% 12-month use |
| Wahner-Roedler[328] | 2005 | cross-sectional survey | 304 | United States | Fibromyalgia patients attending Fibromyalgia Clinic | 37% 6-month use |
| Walker[329] | 2004 | cross-sectional survey | 1913 | Australia | Survey respondents representing adults of voting age from the Australian population with low back pain | 19.3% use |
| Wall[330] | 2007 | cross-sectional survey | 54 | United States | Fibromyalgia patients in urban community base rheumatology clinic 2005 | 48.1% lifetime use |
| Wang[333] | 2004 | cross-sectional survey | 1235 | United States | Surgical patients in urban hospital 2002 | 5.4% current use |
| Wang[334] | 2005 | cross-sectional survey | 950 | United States | Pregnant, low-back pain patients in urban hospital antenatal clinics 2002-2003 | 11.7% use prior to birth, 5.9% use during pregnancy |
| Watanabe[336] | 2015 | cross-sectional survey | 3211 (n=10,400 surveys with response rate 31%) of which 438 used orthopaedic CAM | Japan | General public aged 20 to 69 years registered to internet survey company 2011 | 1.77% 1-month use, 17.8% 1-month use among CAM users |
| Weeks[337] | 2015 | cross-sectional survey | 5422 (n=18,992 or 28X7% response rate) | United States | Members of The Gallup Panel, 18yrs>, 2015 | 13.7% 12-month use |
| Weigel[341]*  And  Weigell[339]  And  Weigell[338] | 2010 | retrospective longitudinal cohort | 5510 | United States | Adults aged 65+; Survey on assets and Health dynamics among the older old (AHEAD), Medicare claims 1993-2007 | 14.6% 15-year use |
| Weigel[338] | 2014 | retrospective cohort | 12170 | United States | Community dwelling Medicare beneficiaries >65 years of age in the United States | 7.4% use spine conditions |
| Whedon[344] | 2016 | retrospective longitudinal cohort | 7502 | United States | Chiropractic patients with Medicare benefits, aged 65 to 99, residing in LA county | 3% 12-month use |
| Whedon[342] | 2011 | retrospective longitudinal cohort | 1759615 | United States | Chiropractic patients with Medicare benefits, aged 65 to 100 | 7.6% 12-month use |
| Whedon[345] | 2012 | retrospective longitudinal cohort | 12192900 | United States | Chiropractic patients with Medicare benefits, aged 65 to 101 | 7.5% 12-month use men, 7.7% 12-month use women |
| Whedon[343] | 2012 | retrospective longitudinal cohort | 1759615 | United States | Chiropractic patients with Medicare benefits, aged 65 to 102 | 31.0% 12-month use back pain, 19.2% 12-month use cranial diagnosis |
| White[346] | 2011 | retrospective longitudinal cohort | 44287 | United States | Military Members Inpatient/outpatient medical services | 8.1% 12-month use |
| Whitlock[347] | 2001 | cross-sectional survey | 2423 | United States | Non HMO members; Portland Oregon | 16.5% 12-month use, 28% lifetime use |
| Wilkinson[348] | 2001 | cross-sectional survey | 300 | Australia | Rural NSW Australian electorate | 18.3% 12-month use, 55% lifetime use |
| Wilson[349] | 2015 | cross-sectional survey | 16546 | United States | Back and/or joint problems, aged 18-85 years | 24.4% 10-year use |
| Wolinsky[350] | 2007 | cross-sectional survey | 4310 | United States | National, aged 70+ years | 4.6% 12-month use, 10.3% 4-year use, |
| Xu[351] | 2007 | cross-sectional survey | 46673 | United States | National MEPS 1996 and 1998, adults | 14.47% 12-month use non-Hispanic Caucasians, 9.46% 12-month use Hispanic, 6.07% 12-month use African American, 3.77% 12-month use Asian, 8.74% 12-month use other |
| Xue[352] | 2008 | cross-sectional survey | 1067 | Australia | General population over the age of 18 years, 2005 | 16.1% 12-months use |
| Yakirevitch[353] | 2009 | cross-sectional survey | 90 | Israel | Chronic rhinosinusitis, aged 18 to 77 years presenting to Medical clinic | 0.33% lifetime use |
| Yang[354] | 2002 | cross-sectional survey | 458 | Taiwan | Chronic liver and gastrointestinal disorders; presenting to a city general hospital | 3.7% lifetime use |
| Yussman[355] | 2004 | cross-sectional survey | 7371 | United States | National, aged 21 years or younger | 0.82% 12-month use, 41.0% 12-month use among CAM users |
| Zhang[356] | 2007 | cross-sectional survey | 1064 | Australia | National | 16.1% 12-month use <65 years old, 15.7% 12-month use >65 years old |
| Zhang[357] | 2008 | cross-sectional survey | 1731 | United States | West Texas population served by university health centre | 22.2% lifetime use, 42.7% lifetime use among CAM users |
| Zodet[358] | 2012 | cross-sectional survey | 22128 | United States | MEPS insurance coverage, National | 5.2% 12-month use |
| Zun[359] | 2002 | cross-sectional survey | 193 | United States | Emergency department patients in Midwest inner city | 12.5% lifetime use |

*Citation used when the combined results are identified throughout this review

**Table 2: Reason for attending chiropractic care, patient demographics, and services provided in relevant studies**

| First author | Year | Study design | Number of patients | Country | Patient population | Reason for attending chiropractic care | Patient demographics | Services provided |
| --- | --- | --- | --- | --- | --- | --- | --- | --- |
| Ailliet[27] | 2010 | cross-sectional survey | 517 | Belgium | Current chiropractic patients | neck and back: 25.3%, low back pain radiating into 1 leg: 21.4%, low back pain: 16.4%, neck radiating into arm: 9.9%, neck pain: 7.6%, interscapular pain: 6.0%, neck pain with headache: 5.0%, headache: 1.9%, leg pain: 1.8%, arm pain: 1.6%, chest pain: 0.4%, other: 2.7% | 54.7% Female, 43 Years (16.4), 70.3% employed | Static palpation: 88%, motion palpation: 85%, analysis/discussion of diagnostic imaging: 80%, visual posture analysis: 72%, orthopedic examination: 64%, neurologic examination: 54%, anamnesis using printed questionnaire: 38%, blood pressure: 18%, abdominal examination: 5%, examination of heart and lungs: 5%, examination of vision and hearing: 5%, posture using instrumentation: 1%  Diversified: 93%, Activator: 41%, Gonstead: 21%, Applied Kinesiology: 19%, Sacro-Occipital Technique: 14%, Specific upper cervical: 10% and Ergonomic advice: 81%, Exercise Therapy: 81%, Trigger point therapy: 79%, ice/cold: 74%, traction: 50%, supplements: 31%, massage: 28%, orthoses: 23%, active exercise in practice: 13%, Cox flexion-distraction: 11%, dietary advice: 43% |
| Alcantara[29] | 2012 | cross-sectional survey | 126 | United States | Pregnant, current chiropractic patients | Primary complaint: Sacro-spinal pain: 79.1%, other nms pain: 10.8%, possible prevention of dystocia: 1.7%, abnormal fetal position: 2.5%, round ligament discomfort: 3.3%, headache: 0.9%, other: 1.7% | 100% Female, 30.56 years | X |
| Alcantara[28] | 2009 | cross-sectional survey | 812 | United States | pediatric, current chiropractic patients | musculoskeletal: 44%, neurological: 12%, ear, nose and throat: 10%, Immune dysfunction: 7%, Colic: 5%, Challenged child: 5%, Constipation: 4%, Enuresis: 3%, Acid reflux: 2%, Asthma: <2%, Other: 6% | 47.0% Female, 6.9 years | X |
| Al-Windi[30] | 2004 | cross-sectional survey | 1433 | Sweden | Adults aged 16 years and above | X | 58.8% Female, 44.8 years | X |
| Astin[31] | 2000 | cross-sectional survey | 728 | United States | Elderly, Blue Shield Medicare members | X | 55% Female | X |
| Bartlett[33] | 2001 | Cross-sectional | 24,676 | United States | General population Non-institutionalised, civilian household population | X | mean age 44.0 (SE 0.95), 58.1% F | 3.0% of visits: x-rays, 3.2% of visits: ultrasound; UNIT OF ANALYSIS IS VISITS: back disorders NEC 25.3%, other cervical spine disorder 11.7%, 5.1% invertebral disc disorder, 7.0% back sprains NEC, mononeuritis of leg sprain 5.9%, joint disorder 3.3%, arthropathies 2.6%, traumatic dislocation 4.9%, injury due to external causes 4.5%, connective tissue disorders of muscle, ligament, fascia 3.3%, curvature of the spine 2.4%, other conditions 14.5%, no condition 9.5% |
| Blum[35] | 2008 | cross-sectional survey | 1316 | United States | SOT patients | Self-care: 40.7%, at risk: 15.7%, sick role: 17.2%, wellness: 14.0%, prevention: 12.4% | 63.6% Female | X |
| Branson[37] | 2009 | retrospective cohort | 2325 | United States | Hospital based chiropractic patients | lumbar spine: 28.8%, cervical region: 24%, thoracic region: 16.5% | 69.4% Female, 42 years (SD 14.86) | X |
| Breen[38] | 1977 | Cross-sectional | 2987 | United Kingdom | Patients from chiropractic clinics | 53.4% lower back, 20.5% neck, 19.9% lower leg, 17.5% thigh and knee, 12.7% head, 12.3% shoulder, 10.8% hip and buttock, 9.5% thorax, 8.4% upper arm and elbow, 6.8% ankle and foot, 4.2% wrist and hand, 3.5% abdomen and groin, 2.5% lower arm, 1.3% not specifically located | 53.1% F; mean age 47.0 M/F | For patients with low back pain (n=1598): 88.7% static palpation, 89.1% movement palpation, 70.8% radiograph, 79.6% orthopedic examination, 74.0% neurological, 74.4% vital systems, 8.3% dipstick urinalysis, 5.9% haemoglobin  For patients with low back pain: 69.1% lumbar manipulation, 50.6% sacroiliac manipulation, 0.5% other joint movements, 16.5% soft tissue and pressure techniques, 2.5% traction, 3.4% electrical treatment, 0.5% heat, cold, chemical applications, 5.8% postural supports, 2.4% exercises, 2.5% vitamins or herbs, 1.3% advice or counselling |
| Bringsli[39] | 2012 | cross-sectional survey | 178 | Denmark | Current chiropractic patients | Maintenance care (41%); non-maintenance care (59%) | Maintenance care patients: 67% Female; non-maintenance care patients: 65% | Maintenance care patients: spinal manipulation: 96%, trigger point therapy: 64%, massage: 38%, advice: 26%; non-maintenance care patients: spinal manipulation: 92%, trigger point therapy: 77%, massage: 54%, advice: 34% |
| Brown[42] | 2014 | cross-sectional survey | 486 | Australia | Current chiropractic patients | Musculoskeletal (68.7%), general health and well-being (21.2%), headaches (5.5%), non-musculoskeletal (4.6%) | Occupation: retired (15.1%), administration (13.2%), trades (20.4%), other (51.3%) | X |
| Brown[43] | 2013 | Cross-sectional | 757 | Australia | General public | X | 48.9% F | X |
| Brunelli[44] | 2003 | Cross-sectional | 180 | United States | Patients with peripheral neuropathy from outpatient clinics | X | mean age 64 | X |
| Bryant[45] | 2003 | retrospective cohort | 1018 | Australia | Teaching clinic, Current chiropractic patients | Lumbar: 40%, cervical: 24%, thoracic: 19%, peripheral: 17% | 46% Female, 36.6 years, 87% employed (white-collar workers 37%, blue-collar workers 31%, unemployed 13%, students 19% | X |
| Bryner[46] | 1996 | cross-sectional survey | 1567 | Australia | Current chiropractic patients | low back: 48%, neck (including headache): 35%, thoracic: 9%, extremity: 7%, Para musculoskeletal: 1%, checkup: 0.7%, stomach-related symptoms: 0.3% | 57% Female, 41 years (median) | X |
| Cambron[47] | 2007 | cross-sectional survey | 163 | United States | Adult patients (categorized into suburban, urban, and university-affiliated | X | Suburban patients (n=73): 49.3% female, 50.5 years old (range 22-83)  Urban patients (n=62): 55.0% female; 55.1 years old (range 21-89)  University-affiliated patients (n=28): 65.4% female; 33.3 years old (range 21-58) | X |
| Carey[50]*  And  Carey[51] | 1995 | cross-sectional survey | 4437 | United States | North Carolina, general population with low back pain | X | 60+ years old: 5%; 37% female, currently employed: 85% | X |
| Carey[48] | 1995 | prospective cohort | 1633 | United States | North Carolina patients with acute low back pain | X | Urban: 40 years, rural: 44 years; Urban: 50% female, rural: 45% female | 67% urban radiography, 68% rural radiography, 8% urban CT or MRI, 7% rural CT or MRI |
| Carey[49] | 1999 | prospective cohort | 921 | United States | North Carolina patients with acute low back pain that resolved within 3 months | X | 82% employed (at 6 months) | X |
| Cherkin[53] | 2002 | cross-sectional survey | 2550 | United States | consecutive visits, collected in 1999 | Back symptoms 44.2%,Neck symptoms 22.5%,Wellness 10.1%,Headache 4.6%,Shoulder symptoms 3.4%  Back symptoms 41.0%,Neck symptoms 24.5%,Wellness 8.7%,Headache 6.4%,Shoulder symptoms 3.9% | Female:  Arizona 58.2%  Massachusetts 57.1%  Age:  <15 years  Arizona 3.7% (0.8)  Massachusetts 2.7 (0.5)  15-64 years  Arizona 76.5%% (1.9)  Massachusetts 85.7 (1.2)  65+ years  Arizona 19.8% (1.8)  Massachusetts 11.6% (1.2)  Occupation: N/A | X |
| Cleary[55] | 1982 | cross-sectional survey | 1026 | United States | Wisconsin, adults 18+ | X | 57.7% Female, 51 years, currently employed (57.3%) | X |
| Conboy[56] | 2005 | cross-sectional survey | 2055 | United States | National | X | Female (53%); age: ≤24 (6.8%), 25-34 (19%), 35-49 (34%), ≥50 (40%); employment: full-time (59%), part-time (8.6%), student (2.5%), homemaker (6.4%), unemployed (1.9%), retired (16%), disabled (4.3%), other (2.2%) | X |
| Cote[57] | 2001 | cross-sectional survey | 907 | Canada | neck pain and/or low back pain | X | Female (48%); age 43.8 (SD 11.6); location of residence: urban (40%), rural (60%), employment: full-time (56.2%), part-time (19.2%), homemaker (13.7%), retired (6.8%), unemployed (4.1%), student (1.4%) | X |
| Coulter[58]*  And  Coulter[59] | 2002 | cross-sectional survey | 1275 | United States and Canada | 10 patients systematically chosen throughout a single day from 131 chiropractors 875 patients in the United States, 400 patients in Toronto, Ontario, Canada | Back-related 76%,Neck/cervical 27%,Low-back 22%, Back or low back sprain/strain, injury, subluxation, pinched nerve, or simply “back” or “low back”: 41%  - Neck sprain/strain, injury, subluxation, pinched nerve, or simply “neck”: 24%  - Upper and lower extremity problems: 13%  - Non-musculoskeletal problems: 6%  - Headache: 4%  - Strains, disks, injury, pinched nerve, site not specified: 4%  - Disc problems: 3%  - Scoliosis or rotated spine: 2%  - Other musculoskeletal problems: 0.4% | United States: 62.5% females; 41.9 years old  Canada: 59.5% female; 42.8 years old Age 42.2, | Diversified (92%), Cox flexion (39%), Nimmo-Tonus (37%), Gonstead (36%), Activator (35%), Patient education (95%), Exercise (92%), Ice therapy (78%), Massage (77%), Electrical therapy (71%), Physical therapy (70%), Ultrasound (67%), Heat therapy (66%), Acupressure (61%), Traction (58%), Therapeutic supports (56%), Nutritional supplements (52%) |
| Coulter[60] | 1996 | secondary analysis of RCT | 414 | United States | US, adults >=65 | X | 69.6% Female, 79.2 years (SD 3.2) | X |
| Davis[61] | 2010 | retrospective cohort | 1871 | United States | 789 adult chiropractic patients in 1997, 1082 Sampling frame ranged from 22,953 (1998) to 32,737 (2003); response rate ranged from 58X3% to 66X7%; adult reported seeing a chiropractor in previous 6 months | X | In 1997: 56.8% female; 46.9 years old; occupation not reported;  Comparison between 1997 and 2006:  mean age 46.9 and 48.0 yrs; female 56.8 % and 59.5% ; | X |
| Dunn[69] | 2006 | cross-sectional survey | 100 | United States | 100 consecutive DC consultation of US veterans | Lumbar spine 82%, Cervical 19%, Thoracic 7%,  Extremities 4% | Female 12%,  Age 55 | X |
| Dunn[68] | 2008 | Cross-sectional | 292 | United States | Patients in Veterans Affairs Medical Centre Chiropractic Clinic | 21.92% cervical, 2.74% thoracic, 71.57% lumbar/sacroiliac, extremity/other 3.77% | 12% F, mean age 54.83 (15.93) | 58.22% combination of HVLA SMT, and either flexion distraction or joint mobilization; 25.34% received only flexion distraction or joint mobilization, 15.07% only HVLA SMT, 1.37% received soft tissue therapy without SMT, flexion distraction, or joint mobilization; 21.23% soft tissue therapy, 1.03% ultrasound, 0.68% cryotherapy |
| Ebrall[71] | 1993 | cross-sectional survey | 2500 | Australia | Patients seeking chiropractic care in Australia | Presenting complaint: back pain (40.56%), neck pain (22.92%), headache (14.2%), shoulder pain (8.00%)  Diagnostic grouping: mechanical low back pain (46.36%); neck and shoulder (17.36%); thoracic (16.76%); neck (16.40%); cervicogenic headache (14.32%) | Female (54% of patient visits); mean age 39.45 (35.6-42.2) | X |
| Eirikstoft[72] | 2014 | prospective cohort | 923 | Denmark | low back pain | X | Female (45%), Mean age (43 years old; SD 12) | X |
| Elder[75] | 2015 | cross-sectional survey | 6068 | United States | Oregon and Washington, patients with chronic musculoskeletal pain | Back pain: 75%, Joint pain: 57%, Arthritis: 54%, extremity pain: 55%, neck pain: 47%, muscle pain: 32%, headache: 26%, fibromyalgia: 16%, abdomen/pelvis" 12%, other: 9% | X | X |
| Ellis[78] | 1994 | cross-sectional survey | 300 | Australia | Current chiropractic patients | low back pain: 48%, cervical and shoulder: 25.7%, thoracic pain: 9.3%, cervicogenic headache: 9%, other: 5%, lower limb: 4.3%, upper limb: 2.7%, visceral: 2.3% | 57% Female | X |
| Fadanelli[85] | 2012 | Cross-sectional | 112 | Italy & Canada | Children with diagnosed rheumatic disease | X | 73% F, mean age 10.1 | X |
| French[104] | 2012 | cross-sectional survey | 4464 | Australia | consecutive DC consultation | Back problem 62.42%,Neck problem 15.46%,Muscle problem 9.83%,Healthmaintenance or preventive care 5.75%,Back syndrome with radiating pain 4.87%Musculoskeletal symptom or complaint 4.96%,Headache 4.05%,Sprain or strain of joint 3.78%,Shoulder problem 1.97%  Nerve-related problem 1.49%,General symptom or complaint 1.15%  Bursitis, tendinitis or synovitis 1.06%,Kyphosis and scoliosis 1.06%  Foot or toe symptoms 1.09%,Ankle problem 1.04%  Osteoarthrosis 0.88%,Hip symptom 0.79%  Leg or thigh symptom 0.79%,Musculoskeletal injury 0.75%  Depression 0.66% | Occupation:  Managers 12.06%  Professionals 21.95%  Technicians and trades workers 9.47%  Community and personal service workers 6.49%  Clerical and administrative workers 8.82%  Sales workers 4.56%  Machinery operators and drivers 2.56%  Labourers 2.68%  Home duties 7.82%  Retired 13.93%  Student 9.07%  Unemployed 0.58% | Manipulation (56.07%),  Soft tissue (50.08%),  Activator (22.64%),  Drop piece (21.49%),  Blocks (18.32%),  Mobilisation (12.41%),  Chiro system (9.28%),  Flexion distraction (3.43%),  Acupuncture (2.39%),  Ultrasound (2.00%) |
| French[105] | 2013 | cross-sectional survey | 7519 | Australia | General medical practice patients who reported they used chiropractic services in last 12 months | X | Female: 67%  Age:  18-34: 16%,  35-54: 50%,  55-76: 34%  Occupation:  Employed/student: 72%,  Not employed 25%,  Unable to work: 3% | X |
| Gaffrey[111] | 2004 | Cross-sectional | 220 | Australia | Women (18+) at least 36 weeks gestation at antenatal clinic | Musculoskeletal problems during pregnancy (80.9%), other during pregnancy (19.1%) | X | X |
| Gaumer[114] | 2006 | cross-sectional survey | 2398 | United States | visited a chiropractor before | X | 65% female; 35% were 35-50 years old, 22.5% were 51-65 years old, 17.5% were >65 years old, 16.3% were 26-34 years old, the rest were ≤25 years old); 18.8% were retied, 8.8% were in health care, 8.8% were in government, 8.5% were retail/wholesale, 8.0% were manufacturing, 6.3% were finance/banking/insurance, 4.8% were utilities/telecommunications, 3.3% were transportation/hospitality | Diagnosis of problem (45%), physical examination (35%), referral for x-ray (35%), health history (31%), laboratory referral (5%), blood/urine sample (3), referral to another provider (3%)  Spinal manipulation (78%), wellness counseling (13%), nutritional counseling (11%), other counseling (11%) |
| Gkolfinopolous[118] | 2003 | Cross-sectional | 1000 | United Kingdom | Low back pain patients in chiropractic practices | 34.2% leg pain, 65.8% no leg pain | mean age 45.7, 46.5% Female | X |
| Greenfield[129] | 2002 | cross-sectional survey | 150 | United Kingdom | Students, first year medical | X | Of the 3 respondents reporting chiropractic use, 2 were male, 1 female | X |
| Hansen[140] | 1997 | Cohort | 1993 n=31,225; 1994 n=31,800 | United States | Group Health Cooperative (HMO) patients | X | 1993: 0-4y F 40%; 5-9y F 60%; 10-14y F 61.8%; 15-19y F 67.7%; 20-24y F 50.9%; 25-29y 60.9%; 30-34y F 58.8%; 35-39y F 62.7%; 40-44y F 63.8%; 45-49y F 61.5%; 50-54y F 47.0%; 55-59y F 57.4%; 60-64y F 45.2%; 65-69y F 54.2%; 70-74y F 73.3%; 75+y F 83.3%; Total F 60.0% 1994: 0-4y F 100%; 5-9y F 80%; 10-14y F 78.9%; 15-19y F 62.0%; 20-24y F 64.1%; 25-29y 55.9%; 30-34y F 59.1%; 35-39y F 63.7%; 40-44y F 58.9%; 45-49y F 60.5%; 50-54y F 50.0%; 55-59y F 53.2%; 60-64y F 65.6%; 65-69y F 77.1%; 70-74y F 47.1%; 75+y F 40.0%; Total F 60.0% | X |
| Hartvigsen[146]*  And  Hartvigsen[147] | 2003 | cross-sectional survey | 1962 (1118); 1999 (1897) | Denmark | Current chiropractic patients | 1962: 51% LOW BACK PAIN, 12% neck pain, 11% headache, 7.5% nonmusculoskeletal disorders. 1999: 53% LOW BACK PAIN, 15% neck pain, 4% headache, 3% nonmusculoskeletal complaints | 51.5% female, mean age 42 (range 0-90) years | X |
| Hawk[149] | 1999 | cross-sectional survey | 563 | United States | Us chiropractors | X | X | Massage (72%), acupressure (72%) |
| Hawk[148] | 2001 | cross-sectional survey | 7651 | United States | Current chiropractic patients | 46% back, 12.7% neck, 10.3% nonmusculoskeletal, 5.5% shoulder, 5.1% maintenance, 4.8% subluxation correction, 2.5% lower extremity, 2.4% hip, 2.2% pain unspecified, 2.1% upper extremity, 1.6% missing/unclassifiable, 1.3% car accident | X | X |
| Hawk[152] | 2000 | Prospective longitudinal | 805 | United States and Canada | Patient’s aged 55 and older, who had not been seen by the DC for the preceding 6 months | Health maintenance 7.5 %, Other 2.5%, Missing 5.7%;  Head 1.2%, Neck 8.5%, Back/Spine 32.9%, Shoulder 3.1%, Arm/elbow/hand 2.4%, hip 5.6%, Knee/ankle/foot 2.7%, Multiple extremity 3.0%, Nonmusculoskeletal 0.8%, Other 0.4%, Multiple locations 35.5%, Missing 3.9%; | Female 60.1,  Age N/A,  Employment 34% | Activator (46.3%), Diversified (27.3%), Gonstead (14.0%), Vitamin mineral and food supplements (84%), Specific dietary practices (62%) |
| Hawk[151] | 1999 | cross-sectional survey | 1511 | United States | seen a chiropractor for any physical problem within the past year | Back 57%,Neck 15%,General spinal 7%,Head 2%,Other musculoskeletal 10%  Non-musculoskeletal 3%,Health maintenance 6% | Occupation:  Working 73.4%  Laid off 1.1%  Unemployed 2.3%  Retired 10.7%  Disabled 1.3%  Housewife 6.9%  Student 3.9% | X |
| Hayden[153] | 2003 | prospective cohort | 54 | Canada | Consecutive pediatric (ages 4 to 18) patients with low back pain | X | Female: 43%  Age: (13.1, 12.1-14.1) | Lumbar facet joint dysfunction/  subluxation (50%)  Sacroiliac joint dysfunction/syndrome (31%)  muscle strain (6%)  disc herniation (1.8%)  spondylolisthesis (1.8%)  Assessment procedures: postural abnormalities (spinal curvation) 22.2%; postural abnormalities (other) 51.9%; spinal subluxation noted 98.2%; abnormal neurologic findings in lower limb(s) 1.9%; pain elicited on orthopedic testing 85.2%; restriction on range of motion testing 79.3%  Manipulation 95.2%  Passive manual therapy 42.9%  Electrical modalities 11.0%  Active management 7.7% |
| Hestbaek[159] | 2014 | prospective cohort | 934 | Denmark | Low back pain in Primary care | X | 45% female, 43 years (34-53) | X |
| Hestbaek[158] | 2009 | cross-sectional survey | 725 | Denmark | Pediatric patients treated during a one month period between September 2007 to September 2008 | Abnormal movement 7%, excessive crying 10%,  general well-being 2%,  asymmetry 5%, disturbed sleep 3%, stomach 8%,  ears 2%, nose/throat <1%, motor development 4%,  musculoskeletal system 36%, headache 7%, asthma/allergy 1%, dizziness/lethargy 1%, concentration/hyperactivity 2%  menstruation <1%  prophylactic examination 7%  other 5% | Female: 46%  Age: 7.61 | X |
| Hodges[164] | 2013 | retrospective cohort | 113 | United States | Patients attending a chiropractic student clinic | msk only 52.2%, non-msk only 12.4%, both msk and non-msk 35.4% | 63% female; mean 28 years (78.8% 21-30 yrs) | X |
| Holt[166] | 2005 | retrospective Cohort | 1004 | New Zealand | Patients presenting to chiropractic teaching clinic | 31.7% low back pain with no referral, 6.4% low back pain with referral, 9.5% thoracic, 13.4% cervical no referral, 3.4% cervical with referral, 12.6% headaches/migraine, 6.2% upper extremity, 2.6% lower extremity, 11.6% no complaint, 2.6% visceral/other | 51.9% F, mean age 32.3 (SD 13.2) | X |
| Hsu[170] | 2008 | cross-sectional survey | 206 | Taiwan | Depression, adults (≥50yrs) hospital based | 89.7% symptom relief, 72.4% increased energy, 37.9% improved heeling of well-being, 13.8% immune system benefits | X | X |
| Humphreys[173] | 2010 | cross-sectional survey | X | Switzerland | Current chiropractic patients | X | X | Used on 76%-100% of patients: 55% diversified, 8% gonstead, 10% drop techniques, 1% activator, 3% applied kinesiology, 4% network, 7% mobilization, 4% active muscle release, 1.3% sacrooccipital techniques, 5% PT modalities, 9% massage, 12% trigger point therapy, 3% rehabilitation techniques, 9% therapeutic exercises, 0.7% nutritional counseling, 14% activities of daily living advice, 0.7% taping/strapping, 7% lifestyle counseling |
| Hurwitz[178] | 1998 | cross-sectional survey | 1916 | United States and Canada | Current chiropractic patients | Minneapolis-St. Paul and Ontario: 75% low back pain, 25% reasons other than low back pain. Portland-Vancouver and Miami: 67% low back pain, 33% non-low back pain. San Diego: 60% LOW BACK PAIN, 40% non-low back pain. | San Diego: 53.9% female, 37.0 (15.4) years old, Portland-Vancouver: 54.9% female, 39.6 (15.7) years old, Minneapolis-St. Paul: 56.6% female, 37.5 (15.3) years old, Miami: 53.2% female, 39.4 (17.9) years old, Ontario: 50.4% female, 36.9 (15.9) years old | Low back pain patients only - San Diego: 56.9% X-rays, 1.9% CT, 3.1% MRI. Portland-Vancouver: 39.6% X-rays, 1.9% CT, 1.5% MRI. Minneapolis-St. Paul: 49.5% X-rays, 4.2% CT, 1.0% MRI. Miami: 70.5% X-rays, 0.0% CT, 2.9% MRI. Ontario: 34.9% X-rays, 0.3% CT, 0.0% MRI.  Low back pain patients only - San Diego: smt 78.9%, nonthrust 73.1%, education 38.5%, other 6.2%. Portland-Vancouver: 90% smt, 85% nonthrust, 29.6% education, 6.1% other. Minneapolis-St. Paul: 79% smt, 78.4% nonthrust, 22.6% education, 2.6% other. Miami: 87.6% smt, 79.5% nonthrust, 32.9% education, 3.8% other. Ontario: 80.8% smt, 53.6% nonthrust, 17.2% education, 1.3% other. |
| Hurwitz[179] | 2006 | cross-sectional survey | 1043 | United States and Canada | General population | X | United States  Female 56.6%,  Age 45.2 (0.7),  Employment 77.3  Canada  Female 53%,  Age 43.4 (0.8),  Employment 79.7 | X |
| Jacobson[183] | 1999 | Cross-sectional | . | United States | Oklahoma Chiropractors | X | X | Elect muscle stim 13.3%, intersegmental traction 11.9%, ultrasound 11.3%, cold therapy 9.6%, hot packs 8.2%, massage 7.8%, diathermy 6.0%, mechanical traction 5.3%, TENS 3.8%, acupuncture 1.8% |
| Jamison[187] | 2002 | Cross-sectional | 147 | Australia | Chiropractic patients | 74% maintenance chiropractic care | F 64.6%; 18-25 years: 6%, 26-45 years: 41%, 46-65 years: 41%, 66-75 years: 6%, over 75 years: 6% | X |
| Jamison[186] | 2003 | Cross-sectional | 782 | Australia | Chiropractic patients | X | 65% F, 46 years or older: 45% | X |
| Jamison[188] | 2005 | Cross-sectional | 627 | Australia | Chiropractic patients (≤18) | X | 0-2 mo: 8.5%; 1-5 y: 27.8%; 6-12 y: 33.3%; 13-18 y: 30.5% | X |
| Jensen[191] | 1989 | cross-sectional study | 604 | Denmark | pediatric patients | Colic=22.6%; spinal=17%; asthma/bronchitis=8.5%; otitis media=8.3%; headache/migraine=6.5% | AGE: categories only <1=38.5%; 1-2=6.8%; 3-6=17.3%; 7-11=17%; 12-16=20.3% | X |
| Kaeser[195] | 2014 | cross-sectional survey | 224 | United States | Teaching clinic, new chiropractic patients | Chief complaint: Low back (31.3%), lower extremity (21.0%), neck (16.5%), mid-back (9.8%), upper extremity (8.5%), headache/facial (7.1%), wellness (1.8%), non-musculoskeletal (1.3%), chest (0.5%) | Female (47.3%), Mean age (37.3 years; range 2 months to 88 years) |  |
| Kalaaji[196] | 2012 | Cross-sectional | 300 | United States | Adult dermatology patients at academic tertiary facility | X | F 53.8% | X |
| Kassak[198] | 1994 | Cross-sectional | X | United States | South Dakota Chiropractors | 47% low back pain, 40.6% neuromusculoskeletal complaint, 12.5% nonneuromusculoskeletal complaints. | X | X |
| Kelner[202] | 1997 | Cross-sectional | 60 | Canada | Chiropractic patients (≥18) | MSK 87%, Headaches 10% | F 58%; Mean age 40 y; Occupational Level: Professional/Managerial 20%, White Collar 68%, Blue Collar 10%, Other 2% | X |
| King[207] | 2004 | cross-sectional survey | 60 | USA | Senior center living independently (African American , Appalachian, white) | Pain management (66%); Symptom control (34%) |  | X |
| Kirby[209] | 2012 | cross-sectional survey | 1310 | Australia | Females with back pain | X | Females aged 55-60 (75% married; EDUCATION ( university: 20%, Diploma/certificate: 21% , high school: 45%, no formal edu: 14% | X |
| Kirby[210] | 2013 | cross-sectional survey | 1310 | Australia | Females with back pain | Pain relief (31.3%); Back pain (27.8%), neck pain (23.4%), leg pain/sciatica (17.3%), headache/migraine (9.9%), arm pain (8%), numbness (7.3%), muscle spasm (7%), weakness, fatigue (5.8%), sleep problem (2.8%), stiffness (2.4%), anxiety/tension (1.8%), instability (1.8%), depression (0.8%) | X | X |
| Kopansky-Giles[212] | 1997 | cross-sectional survey (CCRD, 1995-96) |  | Canada |  | Neuromusculoskel (86.3%), viscero somatic (9.7%), vascular related (2.2%), other (1.8%) | X | Diversified (77.3%), Activator (9.5%), Gonstead (6.6%), SOT (4.1%), Thompson (5.7%), cranio-sacral (1.7%); Electrotherapy (47.3%), Ultrasound (39.2%), cold (57.7%), heat (21.8%), Traction, Flexion distraction (32%); Edu (84.6%: nutritional advise (32.2%), Stress (28.1%), life-style (37.5%); Exercise (70.5%) |
| Krauss[214] | 1998 | cross-sectional survey | 401 | USA | Working age individuals with physically disabling conditions | X | Female (48%), mean age (44 years, s.d: 15.5%), 18-29 (18%), 30-39 (23%), 40-49 (25%), 50 + (31%); EDUCATION: did not finish high school (12), High school (36%), college/trade school (38), graduate school (13%) | X |
| Kronenberg[215] | 2006 | cross-sectional survey | 3068 | USA | Women in four ethnic groups | X | Females aged 18+ (generalized to CAM); EDUCATION: Non-Hispanic Whites less than high school (9.7%), High school (34%), college or more (56.4%), African Americans less than high school (16.2), high school (34.2%), college or more (49.6%), Mexican Americans less than high school (50.6%), high school (27.2%), college or more (22.2%), Chinese Americans less than high school (16.4), high school (21.3$), college or more (62.3%) | X |
| Lafferty[216] | 2004 | cross-sectional survey | 7915 | USA | Insurance claimants with cancer diagnoses | X | Female (63.8%); AGE: 18-40 (43.35%), 41-64 (56.65%) | X |
| Leboeuf-Yde[217] | 2004 | prospective cohort | 951 | Norway | Low back pain | Among patients with persistent low back pain:  - Pain past 2 weeks: constant (45%), often (40%), on and off or rarely (15%)  - Pain pattern past 2 weeks: same intensity (25%), varying intensity (64%), increasing intensity (7%), decreasing intensity (2%), other pattern (1%) | 53% female; 30% 31-40 years old; 24% 41-50 years old; 20% 21-30 years old; 14% 51-60 years old; 6% 61-70 years old; 5% 12-20 years old; 2% 71-80 years old | Treatment at 1st visit:  - Spinal manipulation (95%)  Treatment at 4th visit:  - Spinal manipulation only (15%)  - Spinal manipulation and other treatment (81%)  - Mobilization and other treatment (2%)  -Neither spinal manipulation nor mobilization, but other approach (2%) |
| Leboeuf-Yde[218] | 2005 | cross-sectional survey | 5607 | Denmark | Chiropractic patients | Headache (29%), dizziness (8%), neck problem (51%), arm problem (12%), midback problem (30%), low back problem (60%), sciatica (16%), shoulder problem (21%), pelvic/hip problem (23%), other extremity (12%), non-musculoskeletal problem (8%), maintenance/wellness (16%), subluxation correction/management (16%) (>1 reply possible) | Female (60%); age: 18-24 (9%), 25-44 (43%), 45-64 (36%); 65+ (9%); employment: full-time (41%), part-time (11%), self-employed (15%), homemaker (11%), retired (9%), student (2%), unemployed (2%), other (2%) | Manipulation (83%), soft tissue therapy (52%), mechanically assisted adjustments (35%) |
| Lee[220] | 1985 | cross-sectional survey | 3,686,000 | Canada | Canadians with arthritis, rheumatism, or back, limb and joint disorders | Arthritis, rheumatism, back, limb, joint disorders (16%); "Serious back and spine problems" (4.4%) | Female (18.8%); AGE: >15 (1.3%), 15-64 (17%), 65+ (48.4%);OCCUPATION: Working (16.2%), Housework (30.7%), School (2.5%), Retired for health reasons (13.2%) | X |
| Lee[219] | 2000 | cross-sectional survey | not reported | USA | Average number of patients per week at Boston-area DCs | X | Age: <16 (8%) | Neurologic examination (77%), Radiographic examination (59%), Orthopedic examination (22%), Laboratory tests (8%)  Diversified (62%), Activated (40%), Sacro-occipital (37%) |
| Legorreta[221] | 2004 | cross-sectional survey of administrative claims data | 1,700,000 | USA | Health plan members with and without chiropractic coverage | Neck (12.5%) and Back (22.3%) Pain | Female with chiropractic coverage (51.6%); Mean age with coverage (33%), <17 (32%), 18-21 (5%), 22-35 (15%), 36-55 (34%), 56-64 (8%), 65+ (6%) | X |
| Lewis[222] | 2001 | cross-sectional survey | 416 | Canada | Hamilton-Wentworth medical residents | X | Female (45.7%), Age: <30 (10.4%), 31-50 (38.1%), 51-70 (31.1%), 70+ (17.1%) | X |
| Lim[223] | 2005 | cross-sectional survey | 503 | Australia | Children | X | Mean Age 7.5 (Range: 0-19), <2 (20%) | X |
| Lim[224] | 2006 | cross-sectional survey | 113,229 | Canada | Adults | X | Female (55.76%); Age: 20-34 (24.5%), 35-49 (34.1%), 50-64 (23.5%), 65+ (21.6%) | X |
| Lind[225] | 2009 | cross-sectional survey (claims data) | 237,500 | USA | Insurance claimants | X | Female (57.7%); Urban use (31.8%), Large Town use (44.6%), Small Town use (45.8%), Isolated use (42%) | X |
| Lind[227] | 2006 | cross-sectional survey | 20,722 | USA | Diabetes Patients | Musculoskeletal (79%), Renal disease, retinopathy, CVD, lower extremity ulcers | Female (57%); Median Age: 43 | X |
| Lind[226] | 2007 | cross-sectional survey | 13,792 | USA | Fibromyalgia patients | X | Female (74%), Median Age: 47 | X |
| Lind[228] | 2005 | cross-sectional survey (claims data) | 104,358 | USA | Insurance claimants with back pain | X | Female (59%), Median Age: 49 | X |
| Liow[229] | 2007 | cross-sectional survey | 288 | USA | Epilepsy patients | Epilepsy management (25%) | Education: Less than high school (14%), High School (34%), College (14%), Higher (5%) | X |
| Lishchyna[230] | 2012 | cross-sectional survey | 580 | Canada | New patients who attended the CMCC Bronte Harbour Chiropractic Clinic between July 1, 2006 and July 31, 2008 | cervical 9.9%,Thoracic 5.3%,Lumbar 29.2%,Multiple site 34.6%,Extremity 21.0%  cervical 11.5%,Thoracic 2.1%,Lumbar 21.4%,Multiple site 48.0%,Extremity 17.0%  Duration:  More than one year 42.1%  Up to one month 25.5%  1-6 months 19.2%  6-12 months 13.2% | Female: 57.7%  Age: 43, SD 18  Occupation: professionals 24.4%  Students 16.5%  Skilled workers 12.9%  Retired 11.7%  Unemployed ~10%  Unskilled workers ~11%  Managerial ~5%  Self-employed ~4%  Clerical 3%  Homemaker ~1% | Diagnosis:  Joint sprain 70.0%  Muscle strain 12.6%  Joint and muscle (sprain/strain) 9.3%  Vertebral facture 3.0%  Nerve (cord, nerve root, peripheral) 2.8%  No diagnosis or missing 2.4%  Spinal manipulation 54.7%  Soft tissue therapies 32.1%  Exercise prescription 29.8%  Electro-modalities 12.1%  No treatment 3.8% |
| Lorea[231] | 2007 | cross-sectional survey | 1,445 | USA | Non-institutionalized Mexican Americans | X | Female (62%); Education: 0-6 years (72.2%), 6+ years (27.8%) | X |
| Mackenzi[232] | 2003 | cross-sectional survey | 3,789 | USA | Ethnically diverse adults | X | Female (49%); Age: 18-29 (21%), 30-44 (39%), 45-64 (28%), 64+ (12%); Education: less than high school (16%), High School (29%), college (19%), postgraduate (9%) | X |
| MacLennan[233] | 2006 | cross-sectional survey | 3,015 | Australia | Teens and adults | X | Female (58.4%); Age: 15-24 (16.7%), 25-34 (18.8%), 35-44 (20.2%), 45-54 (18%), 55-64 (13.3%), 65+ (13.1%) | X |
| MacPherson[234] | 2015 | cross-sectional survey | 544 | United Kingdom | Chiropractic patients | X | Female: 66%  Age: 54.5 (IQR 43-71) | X |
| Madsen[235] | 2003 | cross-sectional survey | 622 | Denmark | Paediatric patients | Strengthen immune system (8%), gastrointestinal symptoms (58%), joint symptoms (25%) | X | X |
| Malmqvist[236] | 2008 | cross-sectional survey | 44 chiropractors; number of patients not relevant | Finland | Members of Finnish Chiropractic Union | X | X | Treatment used: Diversified, Activator, Soft tissue. |
| Marchand[237] | 2012 | cross-sectional survey | 19821 | Italy | Pediatric patients (0 to 18 years old) | Skeletal 57.0%,Neurologic 23.7%  Gastrointestinal 12.4%  Infection 3.5%  Genitourinary 1.5%  Immune 1.4%  Miscellaneous 0.5% | Age: Adolescents (13 to 18 years old) 38.4%,  Infants (birth to 23 months) 24.6%,  Children (6 to 12 years old) 23.4%,  Children (2 to 5 years old) 13.6% | X |
| Marrie[238] | 2003 | cross-sectional survey | 20,778 | USA | Multiple Sclerosis patients | X | Female (72.2%), Mean Age: 46.9; Occupation: Employed (40.1%), Unemployed (59.9%) | X |
| Marsh[239] | 2009 | cross-sectional survey | 373 | Canada | Osteoarthritis patients | X | Female (58.7%); Mean Age: 63.2; Education: less than high school (28.4%), high school (30.1%), College or bachelor's (32%), Graduate degree (9.6%); 80+ (15.3%); Employment: Retired (58%), Employed (29.1%), Disability (8.9%), Stay-at-home parent (2.2%) | X |
| Martel[240] | 2005 | cross-sectional survey | 92 | Canada | Children with cancer | X | Female (47%); Age: 0-18 (mean 8.6); 40+ (56%) | X |
| Martin[241] | 2012 | cross-sectional survey | 12,036 | USA | Patients with neck and back problems | X | Female (55.9%); Mean age: 48.9; Education: High school or less (48.4), Any college (51.6%) | X |
| Martinez[242] | 2009 | cross-sectional survey | 500 | Mexico | Adults who have seen a chiropractor | Head and Neck (16.4%), Thoracic (12.6%), Lumbar (29.2%), Pelvis (8.4%), Sacrum and coccyx (3.8%), Extremities (28%), Radiculopathy (0.4%) | Female (61.2%); Age: <20 (7.8%), 20-39 (32.2%), 40-59 (44.2%), 59+ (15.8%); Employment: Housework (26.8%), Blue collar (13.2%), Professional (24%), student (11.4%), shopkeeper (14.8%), other (9.8%) | X |
| McEachrane-Gross[243] | 2006 | cross-sectional survey | 264 | USA | Veterans with cancer or chronic pain | X | Female (6.5%); Mean Age: 65; Employment: Employed (17.6%), Unemployed or unable to work (39.1%), Retired (43.3%); Education: <High school (36.2%), >High school (63.8%) | X |
| McHardy[244] | 2007 | cross-sectional survey | 1,634 | Australia | Amateur golfers | X | Female (19.5%); Mean age 59.2 (s.d. 12.2) | X |
| Metcalfe[245] | 2004 | cross-sectional survey | 400,055 | Canada | Canadian adults with chronic disease (asthma, diabetes, epilepsy and migraine) | X | Female (50.7%), 58.4% married; Education: <high school (26.6%), high school (17.3%), some post-secondary (8.3%), post-secondary completed (47.8%) | X |
| Millar[247] | 2001 | cross-sectional survey | 14150 | Canada | general adult population | X | AGE: 18-24=8%; 25-44=46%; 45-64=34%; 65+=12%; EDUCATION: <high school=18%; high school 15%; postsecondary=29%; diploma/degree=38% | X |
| Miller[248] | 2010 | cross-sectional study | 2,645 | United Kingdom | pediatric patients presenting to chiropractic teaching clinic | 34.7% MSK; 29.6% excess crying; 15.7% feeding disorders | 43% F; AGE: <5=87%; 5-15=13%; | X |
| Mior[249] | 2008 | cross-sectional survey | not reported | Canada | general adult population | X | 2001-2002: 52%F; AGE: categories only: 12-24=15.21%; 25-34=16.51%; 35-44=24.72%; 45-54=20.84%; 55-64=11.68%; 65+=11.04% | X |
| Montalto[250] | 2006 | cross-sectional survey | 848 | United States | general adult population 50 years and over | X | GENDER: 56%F; EDUCATION: <high school=29%; high school=37%; some college=17%; college=17% | X |
| Mootz[252] | 2005 | cross-sectional survey | 2550 | United States | consecutive DC patient visits | Low back pain 41%,Neck/face pain 26%,Extremity condition 9%, Headache 6%,  Wellness 4%,Other 14%  Low back pain 44%,Neck/face pain 23%,Extremity condition 4%, Headache 5%,  Wellness 10%,Other 14% | Arizona  Female 58%,  Age 46.1,  Massachusetts  Female 57%,  Age 44.7 | Arizona  Complete history (23%), Vital signs (18%), Postural examination (38%), Orthopedic examination (41%), Neurological examination (30%), Extremity examination (25%), Spinal examination (79%), Soft tissue examination (56%), Other (2%), X-ray (17%), MRI (1%)  Massachusetts Complete history (9%), Vital signs (6%), Postural examination (26%), Orthopedic examination (30%), Neurological examination (19%), Extremity examination (16%), Spinal examination (80%), Soft tissue examination (56%), Other (5%), X-ray (6%), MRI (1%)  Complete history (9%), Vital signs (6%), Postural examination (26%)  Arizona  Chiropractic adjustment (82%), Cox (4%), Cranial/SOT (5%), Diversified (63%), Gonstead (14%), Thompson (14%), Activator (17%), Ischemic compression (12%), Active release trigger point therapy (10%), Microcurrent (3%), Manual traction (16%), Ultrasound (15%), Electrical stimulation (19%), Hot/cold packs (20%), Acupuncture (6%), Exercise (20%), Counseling/education/self-care (25%)  Massachusetts  Chiropractic adjustment (85%), Cox (10%), Cranial/SOT (6%), Diversified (58%), Gonstead (7%), Thompson (7%), Activator (23%), Ischemic compression (13%), Active release trigger point therapy (14%), Microcurrent (2%), Manual traction (9%), Ultrasound (12%), Electrical stimulation (31%), Hot/cold packs (33%), Acupuncture (0%), Exercise (26%), Counseling/education/self-care (26%) |
| Murthy[253] | 2014 | cross-sectional survey | 1620 | Australia | Australian women aged 60-65 years with back pain, n=1310 | Headache 38%,Nausea 12%,Neck pain 76%,Leg pain 61%,Arm pain 34%,Pins and needles, numbness 33%,Stiffness 50%,Fatigue 26%,Weakness 19%,Depression 20%,Sleeping problems 29%,Instability 7%,Muscle spasm 32%,Anxiety/tension 26% | Female: 100%  Age: N/A  Employment: N/A  Education 9% no formal; 39% high school, 22% trade diploma, 20% university,  90% urban residence | X |
| Nyiendo [261] | 1987 | cross-sectional survey | 2000 | United States | Patients who had not been treated at the college clinic within 2 years | 44% low back pain, 12% headache, 13% check-up, 7% other | Female 53%,  Age 28,  Employment 93%,  Professional/technical/managerial 33%, clerical, sales 22%, services 19%, processing and machine trades 4%, | History, general physical examination, static palpation, leg length check, orthopedic and neurologic tests, and laboratory tests (complete blood count and urinalysis) (100%), Motion palpation (71%), Postural analysis (48%), X-rays (34%)  Spinal manipulative therapy (90%), Soft-tissue therapy (24%), Trigger-point therapy (20%), Ultrasound therapy (13%), Heat application (12%), Cold application (9%), Positive galvanic electrotherapy (3%), Negative galvanic electrotherapy (1%), Sine wave therapy (3%), Exercise instruction (15%), Formal patient education (13%), Orthopedic supports (3%) |
| Nyiendo[263] | 2001 | prospective cohort | 526 | United states | Patients with acute or chronic low back pain as primary complaint | Patients seeking care from chiropractors:  Previous history of low back pain: 89.4%; back pain only (40.9%); pain radiating above the knee (30.7%), pain radiating below the knee (28.4%); baseline pain severity on VAS: 47.7 (SD 24.6); baseline RODQ: 38.3 (SD 15.6) | Female (55.5%); age 42.1 (SD 14.3); employment: full-time (55.8%), part-time (11.6%), self-employed (13.2%), unemployed (19.4%) | Radiographs: 25.6%; CT: 0.4%); MRI 1.5%  Chiropractic manipulation: 83.7%; physiotherapy modalities (48.8%); referral to physical therapist: 0.8%; bed rest: 4.8%; posture advice: 19.6%; supports/braces: 6.7%; nutritional support: 10.6%; self-care education 50.1%; exercise plan: 57.0% |
| Nyiendo[262] | 1989 | cross-sectional survey | 1865 | United States | New patients attending the clinics during 60 consecutive working days (Fall 1986), patients were 18 years or older, who had not been treated at the clinic previously | Percentage of pt. head, neck, upper-/mid-back, low back, extremity, other)  LACC (0, 27, 10, 41, 17, 5)  Palmer (5, 24, 15, 34, 19, 4)  WSCC (4, 20, 14, 37, 19, 6)  Pasadena (0, 19, 11, 39, 19, 14)  Cleveland (5, 25, 12, 31, 22, 5)  Life-West (2, 26, 14, 34, 17, 8) | Female: Cleveland 47%, Palmer-West 51%, Life-West 58%  Age: LACC (36.4, 13.8), Palmer-West (36.0, 13.1), WSCC (37.1, 14.9), Pasadena (42.7, 16.6), Cleveland (37.6, 15.0), Life-West (34.5, 12.6) Occupation: (% employed)  LACC (76%), Palmer-West (76%), WSCC (76%), Pasadena (53%), Cleveland (56%), Life-West (69%)  LACC (28, 25, 23, 24)  Palmer-West (25, 23, 22, 31)  WSCC (41, 29, 17, 13)  Pasadena (41, 20, 19, 20)  Cleveland (63, 18, 11, 8)  Life-West (43, 31, 16, 11) | X |
| Nyindeo[264] | 1988 | cross-sectional study | 217 | United States | paediatric patients at chiropractic teaching clinic | Mid or lower back pain=22.1%; exam only=22.1%; hip/shoulder/extremities=21.6%; other=20.7%; non-MSK=20.2% | 50% female | MSK vs non-MSK: SMT=100% vs 56.8%; STT=22.8% vs 7.9%; trigger point=16.5% vs 2.2%; PT=16.5% vs 10.2%; supplements=15.7% vs 29.5% |
| Pedersen[267] | 1993 | cross-sectional survey | 244 | European | children adults, seniors | LOW BACK PAIN (32%), back + leg (13.9%), mid-back & ribs (5.3%), neck (9.8%), neck & head (2.5%), head (3.7%), ear (1.2%), lower extrem (10.6%), upper extrem (12.7%), infant colic (0.8%), scoliosis (0.8%), asthma/bronchitis, ulcerative colitis, vertigo, runny nose (1.6%), check-up (0.4%) ; Trauma (16%), gradual onset (61.4%) | Female: 53.8%  Age: 40.8 (SD 16.0)  Occupation:  Employed full time 44.5%,  Employed part time 4.7%,  Self-employed 14.1%,  Unemployed 2.4%,  Retired 7.5%,  Unable to work 1.4%,  <16 in school 3.9%,  Child < school age 1.5%,  Housewife 11.6%,  Student full time 5.2%,  Miscellaneous 3.4% | Postural analysis 70.8%,  Static palpation 89.9%,  Dynamic palpation 87.9%,  Orthopedic tests (lumbar) 87.3%,  Orthopedic tests (cervical) 83.1%,  Neural exam – reflexes 78.3%,  Neural exam – sensory 60.8%,  Neural exam – muscle testing 64.6%,  Vital systems – pulse 30.9%,  Vital systems – blood pressure 40.2%,  Vital systems – respiration 13.7%,  Vital systems – temperature 10.0%,  Examination abdomen 21.3%,  Examination heart 16.4%,  Examination lungs 15.8%,  Examination mouth 13.0%  Diversified 68.0%,  Gonstead 37.0%,  HIO 16.6%,  Toggle 22.5%,  SOT 18.0%,  AK 19.5%,  Nimmo 43.0%,  Activator 14.0%,  Pettibon 5.3%,  Pierce-Stillwagon 6.5%,  Toftness 5.1%,  Derived from biomechanical principles 40.2%  1st visit:  Manipulative therapy 91.3%, nonmanipulative therapy 94.8%,  Non-prescription medication 3.2%,  Other substances recommended 5.3%,  Supports/bandages 5.2% |
| Pedersen[268]*  And  Pedersen[269] | 1994 | cross-sectional survey | 1014 | Denmark | All patients treated by the chiropractor who completed a patient case form in 1990 | Headaches/neck-arm 28.5%,  Thoracic/chest 7.3%,  Low back-leg 51.8%,  Upper extremity 3.8%,  Lower extremity 4.4%,  Miscellaneous 4.2%  Duration:  Acute (less than 4 weeks) 46.8%,  Subacute (4 wk to 6 months) 25.1%,  Chronic (>6months) 28.1% | Female: 53.8%; mean age 40.8 (SD 16.0); employment: full-time (44.5%); part-time (4.7%); self-employed (14.1%); unemployed (2.4%); retired (7.5%); disabled (1.4%); home duties (11.6%); child or student (10.6%) | X-rays: 26.5%; laboratory tests: 5.6%  Treatment recorded first visit: manipulative therapy: 91.3%; non-manipulative therapy: 94.8%; non-prescription medication recommended: 94.8%; ergonomic advice: 62%; social/emotional counselling 28%; general health advice 30%; explanation of symptoms 72% |
| Phillips[270] | 1992 | cross-sectional study | 392 | United States | patients presenting to chiropractic teaching clinic | X | Private clinics:  Female (47%); age: 18-19 (4%), 20-29 (32%), 30-39 (32%), 40-49 (17%), 50-59 (8%), 60+ (7%); employment: full-time (71%), part-time (12%), unemployed (16%); job description: manual (34%), non-manual (57%);  Teaching clinics:  Female (55%); age: 18-19 (5%), 20-29 (34%), 30-39 (30%), 40-49 (14%), 50-59 (10%), 60+ (7%); employment: full-time (50%), part-time (22%), unemployed (26%); job description: manual (37%), non-manual (48%) | X |
| Phillips[271] | 1992 | cross-sectional survey | 2257 | United States | Teaching clinic patients and private practice | Private clinics: Duration of low back: < 48 hr (14%), <2 weeks (27%), <2 months (18%), 2 months to <1 year (13%), 1year+ (28%); presence of leg pain: right leg (20%), left leg (19%), both legs (18%); Oswestry disability: minimal (42%), moderate (32%), severe (19%%), very severe (7%)  Teaching clinics: Duration of low back: < 48 hr (4%), <2 weeks (12%), <2 months (7%), 2 months to <1 year (10%), 1year+ (33%); presence of leg pain: right leg (10%), left leg (11%), both legs (17%); Oswesty disability: minimal (55%), moderate (30%), severe (12%%), very severe (3%) | Private clinic vs teaching clinic for LOW BACK PAIN patients: GENDER: 47%F vs 55%F | X |
| Poulsen[275] | 2012 | Retrospective review of records and prospective cross sectional survey | 2000 | Denmark | 20 chiropractic clinics | LOW BACK PAIN=35.1%; LOW BACK PAIN+leg pain=15.5%; neck pain=13%; thoracic/chest pain=11%; other=6% | 38.9 ± 19.2 years | X |
| Robinson[280]*  And  Robinson[279] | 2007 | cross-sectional survey | 459 | Australia | general adult population | X | 73% female | X |
| Rosenfeld[282] | 1987 | cross-sectional survey | 585 | United States | general adult population | X | 35.7% female; AGE: 49.64 | X |
| Rubinstein[284] | 2000 | cross-sectional survey | 833 | Netherlands | New chiropractic patients (not seen in 6 months) | Low back: 47%, neck: 19%, headache: 7%, thoracic: 3%, neck and headache: 3%, lower extremities: 2%, upper extremities: 1%, other nms: 1.6%, non-nms: 1.7%, multiple areas of complaint: 15% | 60% Female, 41 years, 17% unable to work at present | X |
| Ruper[11] | 2000 | cross-sectional survey | 311 | United States | Chiropractic patients aged 65+ who received Wellness care for at least 5 years | 38.3% chronic health problem | 66.9% female, mean 72.9 years old (sd 5.93); Race: White 96.1%, Black 2.65, other 1.2% | 70.4% diversified, 28.3%activator, 21.9% Thompson, 20.6% Nimmo, 17.7% Gonstead, 16.1% AK, 13.5% SOT, 1.6% Grostic  mean annual visits 16.95 (sd 12.53068.2% stretching exercise, 55.6% cardiovascular exercise, 45.3% general diet advice, 41.2% General vitamin-mineral advice, 26.0% Condition-specific dietary treatment advice, 35.0% physical therapy |
| Sandnes[286] | 2010 | prospective cohort | 868 | Denmark & Norway | Chiropractic patients who received Maintenance care | 26% Norway (n=106) and 22% Denmark (n=103)pts on maintenance care; 74% Norway (n= 224) and 28% Denmark (n= 318) pts non-maintenance care | X | X |
| Sawyer[290] | 1984 | cross-sectional survey | 390 | United States | Chiropractic college teaching clinic patients | Chief compliant: low back pain39.2%, neck pain 23.1%, upper back pain 21.1%, leg, knee & ankle pain 11.0%, shoulder, arm & hand pain 6.4%, headache 3.3%, menstrual complaints 1.0%, sinusitis 0.8%, jaw pain 0.5%, migraine 0.3%, facial pain 0.3%, hiatal hernia 0.3%, constipation 0.3%, prostatitis 0.3%, myalgia 0.3%, abdominal pain 0.3%, no information 0.8% | 56.2% female, 32.2 years old female, 32.5 years old male, occupation: 12.5% clerical, 12.4% service-related, 8.4% students | overall mean visits 6.6 (sd=6.96); females mean visits 7.1(sd=7.42), males mean visits 6.1 (sd=6.29) |
| Sawyer[289] | 1984 | retrospective longitudinal cohort | 1986 | United States | Chiropractic college teaching clinic patients | Chief compliant: low back pain 30.7%, neck pain 16.3%, upper back pain 7.8%, shoulder pain 4.6%, headache 4.1%, Upper leg or hip 2.6%, lower leg of knee 2.5%, foot or ankle pain 2.0%, other 29.4% | 54.9% female, 36.6 years old (14.8) | X |
| Sharma[297] | 2003 | Prospective cohort | 1009 | United States | Consecutive patients aged 18 or more with a primary complain of low back pain | Back pain 91.8% | Female: 52.0%  Age: 41.5 (11.68)  Occupation:  Full-time 61.7%  Part-time 10.8%  Self-employed 14.9%  Unemployed 12.7% | X |
| Shekelle[298] | 1991 | retrospective cohort | 5279 | United States | National, under the age of 65 years old | pain, swelling, or injury to the back 42% | 51% female, 16% < 18 years old, 70% between 18-50, 14% 51+ years old | First visit: 39% manipulation, 23% physical medicine, 19% office visits, 17% X-rays Repeat visit: 66% manipulation, physical medicine, office visits, and X rays 32% |
| Simon[301] | 2003 | cross-sectional survey | 2550 | United States | Mental health use among patients who sought Chiropractic care | 0.3% mental health concern as primary reasons for visit, 1.2% mental health concern as any reason for visit | X | X |
| Sorensen[305] | 2006 | cross-sectional survey | 1595 | Denmark | chiropractic patients presenting to chiropractic clinics over random one week intervals in 2002 | Lower back or pelvis 49% | X | X-ray (27%) |
| Stano[308] | 1993 | retrospective longitudinal cohort | 395641 | United States | National, with neuromusculoskeletal ICD-9 codes | X | 54.5% female, 38.9 years old | X |
| Stano[309] | 2002 | retrospective longitudinal cohort | 2263 | United States | Low back pain patients | X | 49.9% female, 41.4 years old (12.8) | 31% manipulation, 9.77% hot/cold pack, 8.99% office visit, 7.86% electric stimulation, 6.18% office outpatient visit, 5.16% massage |
| Stevans[311] | 2012 | retrospective cohort | 1260 | United States | Adults 18 years and older from public files for Medical Expenditure Panel Survey (MEPS) 10, 11, and 12 (2005-2008) | Back 46.2%  Neck 14.8%  Extremity 16.5%  Musculoskeletal unspecified 15.9%  Nonmusculoskeletal conditions 6.6% | female: 61.8%  Age:  18-44 (41.6%)  45-64 (40.8%)  65+ (17.6%) | X |
| Stevens[314] | 2005 | cross-sectional survey | 118 of 250 surveyed | United States | US residents | 80% back pain, 7% headaches, 7% leg pain, 3% arm pain, 12% other | Female 60%; 18-25 yrs 6%, 26-34 17%, 35-44 17%, 45-54 29%, 55-64 23%, 65>9%; occupation: 14% homemaker, 11% laborer, 26% professional, 26% retired, 20% skilled, 3% student | 71% adjustment, 10% therapy, 16% massage |
| Stevens[313] | 2007 | cross-sectional survey | 240 | United States | New patients who were 18 years or older, | Low back 62%,  Neck 41%,  Mid back 6%,  Headache 10%,  Upper extremity 33%,  Lower extremity 29%,  Maintenance 3%,  Other 7% | Female: 60%  Age:  18-29 (38%)  30-39 (16%)  40-49 (18%)  50-59 (15%)  60+ (12%) | Home treatments:  Heat 49%,  Ice 26%,  Medication 42%,  Topical 22%,  Exercise 8%,  Other 6%, |
| Stevens[312] | 2007 | Chart review | 256 | United States | Adult patients of the ethnic poor in the East side of Buffalo, NY | Chief complaints: lumbopelvic (57%), neck (18%), thoracic (7%), knee (5%), shoulder (4%), maintenance (2%), headache (1%), ankle (1%), foot (1%), other (1%) | 65% female; 26% 40-49 years old, 25% 50-59 years old, 23% 30-39 years old, 14% 18-29 years old, 13% ≥60 years old | X |
| Suleman[317] | 2001 | cross-sectional survey | 183 | Canada | Low-income patients at urban health clinic | X | 33% female, 37.8 years old; Occupation (n=189): Laborer 14%, student 4%, unemployed 3%, driver 2%, social services 1%, other 10%, not reported 66%; | X |
| Till[320] | 1999 | cross-sectional survey | 262 | South Africa | Teaching clinic patients | spinal 60.2%, head 1.2%, extremity 29.8%, visceral 8.6%, uncertain 0.2% | 79% female; mean age 53.3; occupation: 30.2% pensioners, 19.1% unskilled workers, 12.2% unemployed, and 10.7% housewives | X |
| Waalen[327] | 2005 | retrospective cohort | 453909 | Canada | Current chiropractic patients, Year end PMP data on all chiropractic patients between April 1, 2000 and March 31, 2001 | Cervical 17%,Thoracic 9%  Lumbar 34%,Non-spinal 8%  Multiple sites 26%,Other codes 6%  Female:  Cervical 23%,Thoracic 9%  Lumbar 26%,Non-spinal 7%  Multiple sites 30%,Other codes 5% | Female:53.2%  Age:  less than 20: 14% male, 12% female  20-34.9: 19% male, 21% female  35-49.9: 34% male, 34% female  50-64.9: 22% male, 21% female  65 and over: 11% male, 12% female | Diversified 85%  Activator 7%  Thompson 2.7%  Gonstead 2.4%  Applied Kinesiology 2%  Modalities 29%  Acupressure/trigger point therapy 17%  Exercise – corrective therapeutics 17%  Massage therapy 13%  Acupuncture with needles 9%  Mobilization therapy 4% |
| Waalen[326] | 1994 | cross-sectional survey | 15174 | Canada | New patients who attended all CMCC teaching clinics between 1986 and 1990 | Cervical 32.4%  Thoracic 11.2%  Lumbar 24.4%  Sacroiliac 10.0%  Non-spinal articulations 18.8%  Other 3.2%  Duration:  < 1 month:  Cervical 28%  Lumbar 36%  Thoracic 22%  Sacroiliac 29%  Non-spinal 31%  Other 27%  1-24 months:  Cervical 32%  Lumbar 36%  Thoracic 26%  Sacroiliac 32%  Non-spinal 37%  Other 31%  > 2 years:  Cervical 41%  Lumbar 42%  Thoracic 31%  Sacroiliac 39%  Non-spinal 43%  Other 32% | Female: 49.5%  Age: male 32.5, SD 14.2  Female 33.4, SD 15.0  Occupation: students 26.2%  Clerical 17.4%  Semi-professional 10.4%  Managerial 9.4%  Professional 7.7%  Unskilled 6.7%  Skilled 5.3%  Unemployed 5.2%  Semi-skilled 4.5%  Retired 4.2%  Homemaker 2.9%  Farmer 0.1% | X-ray 34.4% |
| Walker[329] | 2004 | cross-sectional survey | 1913 | Australia | Survey respondents representing adults of voting age from the Australian population with low back pain | X | Female: 57.7%  Age: 42.3 (16.2)  Occupation:  Full-time 51.9%  Part-time 16.9%  Unemployed 3.9%  Home duties 14.3%  Student 2.6%  Retired 10.4% | X |
| Walsh[331] | 1992 | cross-sectional survey | 310 | Australia | Chiropractic teaching clinic | 33.2% low back, 23.6% cervical, 16.5% thoracic, 6.4% shoulder, 1.9% elbow, 1.0% wrist, 0.6% hip, 2.9% knee, 2.6% ankle, 8.1% headache, 3.2% other visceral | 52.6% female, 34.4 (13.5) years of age, occupation: 5.5% heavy manual, 32.3% light manual, 54.4% non-manual, 8.0% unemployed | X |
| Walsh[332] | 1992 | Cross-sectional | 422 | Australia | Chiropractic patients | Clinic A: LOW BACK PAIN 46.2%, Cervical 24.1%, Thoracic 10.2%, Shoulder 4.7%, Headache 4.7%, Other Extremity 4.6%, Other Visceral 6.3%; Clinic B: LOW BACK PAIN 40.3%, Cervical 24.6%, Thoracic 11.4%, Shoulder 3.6%, Headache 6.1%, Other Extremity 2.6%, Other Visceral 6.1%; Clinic C: LOW BACK PAIN 40.5%, Cervical 26.5%, Thoracic 7.0%, Shoulder 10.0%, Headache 6.5%, Other Extremity 8.0%, Other Visceral 1.5%; Teaching Clinic: LOW BACK PAIN 33.2%, Cervical 23.6%, Thoracic 16.5%, Shoulder 6.4%, Headache 8.1%, Other Extremity 9.0%, Other Visceral 3.2% | F: Clinic A 48.2%; Clinic B 48.2%; Clinic C 47.0%, Teaching Clinic 52.6%; Mean Age: Clinic A 33.6 (14.4); Clinic B 33.9 (19.2); Clinic C 34.8 (14.0); Teaching Clinic 34.4 (13.5); Occupation: Clinic A--Heavy Manual 12.0%, Light Manual 34.3%, Non-manual 37.0%, Non-Employed 16.7%; Clinic B--Heavy Manual 4.4%, Light Manual 20.2%, Non-manual 38.6%, Non-employed 36.8%; Clinic C Heavy Manual 21.5%, Light Manual 44.5%, Non-manual 17.5%, Non-employed 16.5%; Teaching Clinic--Heavy Manual 5.5%, Light Manual 32.2%, Non-manual 54.4%, Non-employed 8.0%. | X |
| Ward[335] | 2015 | cross-sectional survey | 44 | United States | Non-Hispanic black adult chiropractic patients from 20 chiro offices across 2 states, | 46.5% back pain; neck pain 18.6%, personal injury18.6% | 54.5% female; 2.3% 18-29yrs, 40.9% 30-49, 56.8% >=50 years old; employed 61.9% | 38.7% diet, physical activity 59.1% advice |
| Weeks[337] | 2015 | cross-sectional survey | 5422 (n=18,992 or 28X7% response rate) | United States | Members of The Gallup Panel, 18yrs>, 2015 | X | Use of Chiro within 12 months: female 57.9%, 24.2% aged 18-34, 30.1% aged 35-49, 27.7% aged 50-64, 18.1% aged 65+, mean age: 48.3 (15.5) years, Employment: 59% full time, 9.1% part time, 3.4% student, 6.5% homemaker, 3.1% not employed, 18.9% retired, | X |
| Weigel[341]*  And  Weigell[339]  And  Weigell[338] | 2010 | retrospective longitudinal cohort | 5510 | United States | Adults aged 65+; Survey on assets and Health dynamics among the older old (AHEAD), Medicare claims 1993-2007 | X | 60.3% female; 76.0 years (4.87) | X |
| Weigel[338] | 2014 | retrospective cohort | 12170 | United States | Community dwelling Medicare beneficiaries >65 years of age in the United States | X | Female (58%), Age: 65-69 (28%), 70-74 (29%), 75-79 (23%), 80-84 (14%), 85+ (7%) | X |
| Whedon[344] | 2016 | retrospective longitudinal cohort | 7502 | United States | Chiropractic patients with Medicare benefits, aged 65 to 99, residing in LA county | X | Asian 11.9%; Black 1.1; Hispanic 0.6; Other/unknown 14.4; White 72.0 | X |
| Wolinsky[350] | 2007 | cross-sectional survey | 4310 | United States | National, aged 70+ years | X | African Americans (AOR = 0.239, p < .001)  and Hispanics (AOR = 0.454, p < .05)  less likely to use than Whites; veterans less likely to use | X |
| Xue[352] | 2008 | cross-sectional survey | 1067 | Australia | General population over the age of 18 years, 2005 | Back pain 65.7%,  Shoulder pain 5.3%,  Neck pain 20.7%,  Headache and migraine 9.3%,  Non-specific musculoskeletal problem 14.9%,  General health and well-being 32.2%,  Improving ability to undertake daily activity 36.2%,  Improve sporting performance 9.4% | Gender:  Female 17.1%  Male 15.0%  Age:  18-34 (15.0%, 11.2-18.9)  35-64 (16.7%, 13.6-19.9)  65+ (15.7%, 10.5-21.0)  Occupation:  Employed (16.8%)  Unemployed/not in work force (14.5%) | X |
| Zodet[358] | 2012 | cross-sectional survey | 22128 | United States | MEPS insurance coverage, National | X | odds of ≥ 1 visit approx. 46% less for Asians, 63% less for Hispanics, and 73% less for blacks compared with whites; use rates were higher for persons 18 to 64 years old with private insurance coverage (5.9%) and those 65 years and older with Medicare (5.4%) compared with  persons 18 to 64 years old with only public (3.1%) or no insurance coverage (3.0%); women were more likely to receive care (5.7% vs 4.6% for men) as were married persons (6.1% vs 4.1% for unmarried persons); Women were more likely to receive chiropractic care  (5.7% vs 4.6% for men) as were married persons (6.1% vs  4.1% for unmarried persons) | X |

*Citation used when the combined results are identified throughout this review

X Not reported
